# Glial Dysfunction and Memory Impairments in a Model of Pediatric Obstructive Sleep Apnea

**DOI:** 10.1002/glia.70230

**Published:** 2026-09-18

**Authors:** Michael R. Williamson, Jorge Botas, Mahyar J. Hedayatpour, Thea Zlatkov, Akdes Serin Harmanci, Christopher J. Yates, Kenneth Nobleza, Roshan Ailani, Adam C. Adler, Farrah Kheradmand, Hyun Kyoung Lee, Benjamin Deneen, Hyun‐Hwan Jeong, Arvind Chandrakantan

**Affiliations:** ^1^ Program in Neurosciences and Mental Health The Hospital for Sick Children Toronto Ontario Canada; ^2^ Department of Physiology University of Toronto Toronto Ontario Canada; ^3^ Data Science Center Jan and Dan Duncan Neurological Research Institute, Texas Children's Hospital Houston Texas USA; ^4^ Department of Pediatrics Baylor College of Medicine Houston Texas USA; ^5^ Jan and Dan Duncan Neurological Research Institute Texas Children's Hospital Houston Texas USA; ^6^ Department of Anesthesiology Perioperative and Pain Medicine, Texas Children's Hospital Houston Texas USA; ^7^ Department of Anesthesiology Baylor College of Medicine Houston Texas USA; ^8^ Department of Neuroscience Baylor College of Medicine Houston Texas USA; ^9^ Center for Cell and Gene Therapy Baylor College of Medicine Houston Texas USA; ^10^ Department of Neurosurgery Baylor College of Medicine Houston Texas USA; ^11^ Department of Pediatrics Texas Children's Hospital Houston Texas USA; ^12^ Translational Biology and Molecular Medicine Program Baylor College of Medicine Houston Texas USA; ^13^ Department of Medicine Baylor College of Medicine Houston Texas USA; ^14^ Dan L. Duncan Comprehensive Cancer Center Baylor College of Medicine Houston Texas USA; ^15^ Department of Pathology and Immunology Baylor College of Medicine Houston Texas USA; ^16^ Center for Translational Research in Inflammatory Diseases Michael E. DeBakey VA Houston Texas USA; ^17^ Biology of Inflammation Center Baylor College of Medicine Houston Texas USA; ^18^ Center for Cancer Neuroscience Baylor College of Medicine Houston Texas USA

**Keywords:** astrocyte, cognition, glia, microglia, neurogenesis, oligodendrocyte, sleep apnea

## Abstract

Pediatric obstructive sleep apnea (POSA) is a common childhood disease that often causes aberrant brain development and cognitive deficits. The pathophysiological underpinnings of cognitive impairments in POSA remain unclear. Here, we examined cellular and molecular aspects of pathology in a mouse model of POSA that features learning and memory deficits. We performed single‐nucleus RNA‐sequencing (snRNA‐seq) of the hippocampus to examine gene expression changes in an unbiased and cell type‐specific manner. This dataset revealed a striking perturbation of transcriptomes across all brain cell types, particularly within glia and neural stem cells. We validated reduced expression of several differentially expressed genes at protein level: QDPR and SOX8 in oligodendrocytes, LRRK2 and NDUFS4 in neural stem cells, TFE3 in microglia, and GLUT1 in astrocytes. Comparison of oligodendrocyte gene expression changes with proteomic datasets suggested impairments in myelination, which we confirmed in vivo. Furthermore, cellular level analyses demonstrated aberrant morphology of oligodendrocytes, astrocytes, and microglia in the hippocampus, and diminished numbers of neural stem cells in the subgranular zone. Our study identifies cellular and molecular glial cell dysfunction in POSA, validates gene targets for further study, and provides an snRNA‐seq dataset to facilitate further data‐driven hypothesis generation.

## Graphical abstract

- Generation and analysis of an snRNA‐seq atlas of the hippocampus in POSA.
- Validation of reduced protein expression of six genes across four cell types.
- Cellular‐level investigation of aberrant glial function in POSA using transgenic reporter mice.

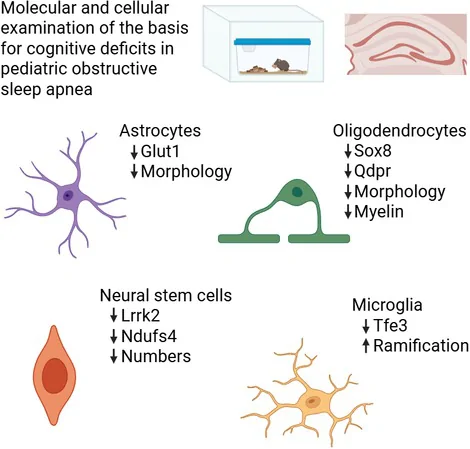

## 1 Introduction

Disordered breathing or obstructive apnea during sleep occurs in approximately 7.5% of children (Chan et al. 2004; Lumeng and Chervin 2008; Xiao et al. 2023; Yang et al. 2024). Pediatric obstructive sleep apnea (POSA) involves frequent upper airway obstruction resulting in intermittent hypoxic periods associated with fragmented and reduced sleep. Children with POSA often have cognitive deficits and neurodevelopmental delay, including impairments in learning and memory, school performance, and behavioral regulation (Chan et al. 2014; Chandrakantan et al. 2020; Xiao et al. 2023). However, the cellular and molecular neural basis for cognitive impairments in POSA has not been examined. Importantly, frontline treatment of adenotonsillectomy fails to restore learning and memory deficits (Baugh et al. 2011; Marcus et al. 2013). Therefore, an improved understanding of the lasting brain pathology resulting from POSA is needed in order to better understand the nature of these deficits and instruct treatment strategies.

In our prior polysomnographic studies of children with POSA we found that reduced oxygen saturation is linked with cognitive deficits (Chandrakantan et al. 2020, 2025). These data suggest that intermittent hypoxia during obstructive episodes drives pathology and cognitive impairments in human patients. Therefore, we recently developed a mouse model of POSA based on human polysomnographic data and cross‐species comparison of oxygen saturation curves (Chandrakantan et al. 2025). This neurodevelopmental model uses forced intermittent hypoxia during light hours beginning in pre‐weaning‐age mice to model hypoxic episodes of POSA. Importantly, this model recapitulates learning and memory deficits, making it a potentially useful preclinical model for studying the neural basis for cognitive deficits in POSA.

Here, we sought to define molecular and cellular changes in POSA to gain insight into disease pathophysiology and identify potential intervention approaches. We performed single nuclei RNA‐sequencing in our intermittent hypoxia mouse model of POSA, generating a brain cell type‐specific transcriptional atlas of the disease. From this dataset, we observed widespread gene expression perturbations across cell types. We present an in‐depth analysis of cell type‐specific gene expression changes and transcriptional regulatory mechanisms in POSA, focusing on the prominent dysfunction of oligodendrocytes, astrocytes, microglia, and neural stem cells. Furthermore, we validate protein level reductions in expression of six candidate genes across these four cell types. Finally, using cell‐type specific labeling with transgenic mouse lines, we demonstrate aberrant cellular morphology of oligodendrocytes, astrocytes, and microglia, and reduced numbers of hippocampal neural stem cells. Together, this study highlights dysfunction of glial cells as a potential cause of lasting cognitive impairments in POSA, identifies molecular targets for further study, and provides a transcriptomic dataset useful for understanding brain molecular changes in POSA.

## 2 Methods

### 2.1 Mice

All animal work was done in compliance with US Department of Health and Human Services, NIH guidelines, and Baylor College of Medicine Institute Animal Care and Use Committee guidelines and protocols. Mice were housed in a 12‐h light–dark cycle environment with food and water freely available. Both male and female mice on the C57BL/6 background were used. We used wildtype mice and the following transgenic mouse lines: *Nestin‐CreER* (Jackson Labs, #016261; for neural stem cell‐specific expression of CreER), *NG2‐CreER* (Jackson Labs, #008538; for oligodendrocyte lineage‐specific expression of CreER), *Aldh1l1‐GFP* (bacterial artificial chromosome line; for astrocyte‐specific expression of GFP), and Ai14 (Rosa‐CAG‐LSL‐tdTomato; Jackson Labs, #007914; crossed with CreER lines for Cre‐dependent expression of tdTomato). For induction of Cre‐mediated recombination, mice were injected (IP) with 100 mg/kg of tamoxifen dissolved in sterile corn oil for 5 consecutive days from P12 to P16.

### 2.2 Intermittent Hypoxia Model of POSA

POSA was modeled with an intermittent hypoxia regimen from postnatal day P14–P70. Cages containing P14 mice and parents were placed into a BioSpherix hypoxia chamber. We used an intermittent hypoxia regimen during sleep hours to reduce oxygen saturation to levels translatable to those seen in human POSA patients, as previously described (Chandrakantan et al. 2025). This was accomplished by cycling the chamber between 21% oxygen (room air) and 10% oxygen. During each cycle, oxygen concentration was reduced to 10% for 2 min and 15 s such that mice spent 2 min at 70% SpO_2_, followed by 45 s for insufflation of room air, and 3 min in room air (Chandrakantan et al. 2025). This 8‐min sequence was repeated 7.7 times per hour during light hours to induce intermittent hypoxia that mimics an apnea hypopnea index seen in human POSA patients. Intermittent hypoxia began at P14 to match the human equivalent age of the youngest patients in human POSA cohorts (Marcus et al. 2013; Chandrakantan et al. 2020). At P23 mice were weaned and separated by sex, then placed back in the chamber until P70. This model primarily mimics hypoxic periods during obstructive sleep events and also results in reduced total amount of time asleep (Chandrakantan et al. 2025), as is seen in human patients (Don et al. 2024). This model is based on previously described human polysomnographic data and mouse oxygen saturation curves with cross‐species translation (Chandrakantan et al. 2025). Control cages were left in chambers with constant room air.

### 2.3 Pattern Separation Behavioral Task

**FIGURE 1:**
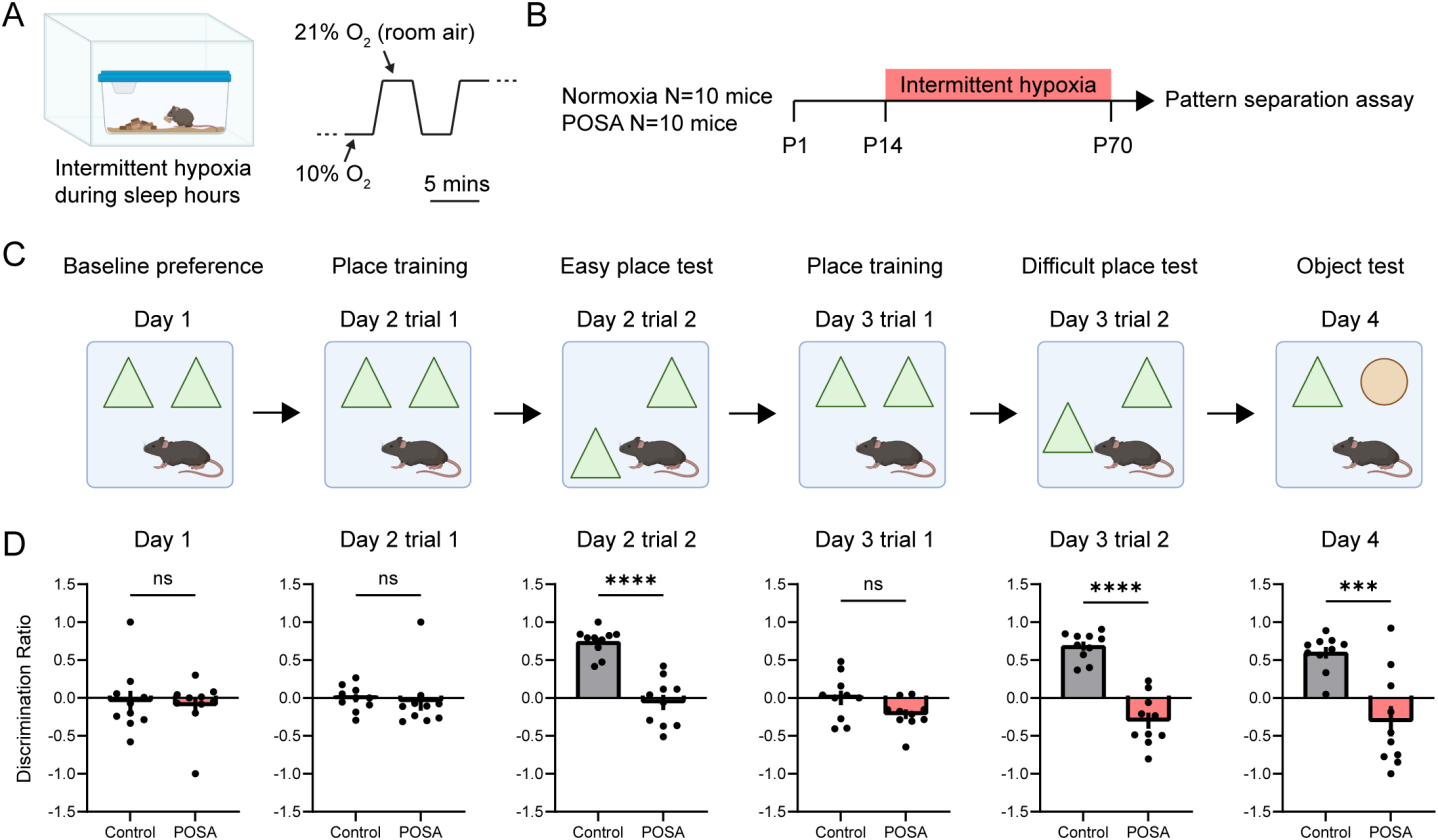
An intermittent hypoxia model of POSA recapitulates learning and memory impairments in mice. (A) Schematic of experimental setup for the intermittent hypoxia model of POSA. Cages were placed inside of a chamber where oxygen concentration was cycled as depicted on the right. (B) Experimental timeline for examining learning and memory behaviors with a pattern separation assay following POSA (intermittent hypoxia) or control (normoxia). (C) Diagram illustrating objects and positions during each trial of the pattern separation assay. Test trails were Day 2, Trial 2; Day 3, Trial 2; and Day 4. (D) Object discrimination during each trial. Graphs correspond to the trial depicted by the diagram above in Panel C. ***p = 0.0005, ****p < 0.0001, unpaired two‐tailed *t*‐tests, *n* = 10 mice/group.

Hippocampus‐dependent learning and memory function was tested using a spatial pattern separation test (van Goethem et al. 2018). This test took place over four consecutive days beginning on P70. During each trial mice were individually placed into a 22 cm × 44 cm plexiglass chamber cleaned with 70% ethanol. The chamber was surrounded on three sides by a white screen, with the fourth side a consistent background to provide spatial orientation. Each session involved two objects placed in predefined locations. All sessions were recorded with a camera positioned directly above the center of the arena to ensure unobstructed tracking. Time spent interacting with each object was quantified offline using ANY‐maze software (Stoelting, v7.6). Animal location, movement, and object exploration were automatically recorded and quantified using ANY‐maze automated animal tracking with standard settings. Object exploration was defined as the mouse directing its nose toward the object at a distance of ≤ 2 cm. Time spent interacting with objects was automatically quantified from ANY‐maze tracking data with consistent settings across all subjects. A discrimination index was calculated as the time spent interacting with the test object (object that was moved or replaced) relative to the total time interacting with either object. A schematic of the test design is shown in Figure 1. One trial was conducted on the first day as a control trial: two identical objects were placed in adjacent quadrants and the time spent interacting with each was measured. On the second day, Trial 1 (training) involved two identical objects placed in adjacent quadrants and the time spent interacting with each was measured. One hour later, Trial 2 (test) involved moving one of the objects to the open adjacent quadrant and the time spent interacting with each was measured. Mice normally interact more with the object that was moved, demonstrating an ability to remember and discriminate object locations (Sivakumaran et al. 2018). Trials 1 (training) and 2 (test) on the third day were identical to those of the second day except the object was moved to an intermediate distance in Trial 2 to provide a more challenging discrimination task. Finally, the fourth day involved a single trial where one object was replaced with a novel object of similar size to the objects used in all prior trials, providing a test of object discrimination.

### 2.4 Single Nuclei RNA‐Sequencing

Hippocampi and surrounding white matter from control and POSA mice (*n* = 3/group) were microdissected and processed for single nucleus gene expression library preparation. Tissue was dissociated and nuclei were extracted using a Nuclei Isolation Kit (Miltenyi Biotec, Cat. #130‐128‐024) and debris removal was performed using a debris removal kit (Miltenyi Biotec, Cat. #130‐109‐398). The quantity and quality of the nuclei were accessed by Acridine Orange and Propidium iodide dye on a Cellometer Auto 2000 (Nexcelom). About 30,000 nuclei were loaded on a 10× Chromium X using Chromium GEM‐X Single Cell 3′ Reagent Kits v4 (10× Genomics, CG000731). After nuclei partitioning and GEM generation, reverse transcription was performed, and cDNA was pooled and further amplified for 12 cycles. The quality of cDNA was assessed by High Sensitivity Tapestation (Agilent Technologies Inc., D5000) and quantified by Qubit 2.0 DNA HS assay (ThermoFisher, Q32851). Final library was then constructed based on the manufacturer's protocol with 11 PCR cycles. Equimolar pooling of libraries was performed based on QC values and sequenced on an Illumina NovaSeq X Plus with a read length configuration of 150 PE targeting for 800 M total reads per sample (400 M in each direction). Nestin‐CreER mice were used for single nuclei RNA‐sequencing to facilitate computational identification of neural stem cells by Cre RNA expression.

### 2.5 Cell Type Proportion and Abundance Analysis

To assess whether POSA was associated with changes in the relative abundance of specific cell types, we performed cell type proportion analysis using the scProportionTest framework (v0.1.2) (Miller et al. 2021). Proportion differences between POSA and control conditions were evaluated based on the annotated cell type labels derived from the single‐nucleus RNA‐seq analysis.

Statistical significance of differences in cell type abundance was assessed using a permutation‐based testing strategy. Briefly, nuclei were grouped by experimental condition and cell type, and observed differences in cell type proportions between POSA and control samples were compared against a null distribution generated by permuting condition labels across nuclei. A total of 1000 permutations were performed to estimate empirical *p* values.

In addition, confidence intervals for the magnitude of proportional differences were estimated using bootstrap resampling (1000 bootstrap iterations). Cell types were considered to exhibit significant changes in abundance if they met both statistical and effect size criteria, defined as a permutation‐based adjusted *p* value < 0.05 (Benjamini–Hochberg correction) and an absolute observed difference in cell type proportion greater than 0.5. This approach accounts for variability in cell counts across samples while avoiding parametric assumptions about underlying distributions.

### 2.6 Differential Gene Expression and Pathway Enrichment

Differential gene expression analysis was performed in a cell type–resolved manner using the Seurat framework. Following clustering and cell type annotation, POSA and control nuclei were compared independently within each cell type cluster to avoid confounding effects driven by changes in cellular composition.

Differential expression testing was conducted using the FindMarkers function in Seurat (Stuart et al. 2019) with the MAST hurdle model (MAST v1.20.0) (Finak et al. 2015), which is well suited for sparse single‐nucleus RNA‐seq data. For each cell type cluster, POSA and control nuclei were contrasted. Positive log2 fold change values indicate higher expression in POSA relative to control.

To account for technical and biological sources of variability, the MAST model included the following latent covariates: sample identity, number of detected genes per nucleus, total UMI count, and mitochondrial transcript fraction. No pre‐filtering was applied at the model fitting stage based on expression prevalence or fold change (log‐fold change threshold = 0; minimum expression fraction = 0), allowing all expressed genes within each cluster to be evaluated.

*p* values were adjusted for multiple testing using the Benjamini–Hochberg procedure as implemented in Seurat. For downstream analyses and visualization, genes were classified as significantly differentially expressed if they met both statistical and effect size criteria: adjusted *p* value < 0.05 and absolute log2 fold change > 0.25.

For pseudobulk differential expression, to assess differential expression at the biological‐replicate level, raw UMI counts were summed across nuclei within each biological replicate for each broad cell type and, separately, each annotated subcluster, yielding up to six pseudobulk profiles (three control and three POSA) per population. Profiles derived from fewer than 10 nuclei were excluded, and only populations retaining at least two biological replicates per condition were tested. Differential expression was assessed using edgeR quasi‐likelihood *F*‐tests following TMM normalization and filterByExpr and, independently, using DESeq2 Wald tests. Because each pseudobulk profile corresponds to one biological sample, both models used condition as the sole design term. A processing sub‐batch was partially confounded with condition and could not be estimated separately. Benjamini‐Hochberg adjusted *p* values were calculated, and genes with FDR < 0.10 were considered pseudobulk significant. Concordance with the cell‐level MAST analysis was quantified using Spearman correlations of log2 fold changes and direction agreement among MAST‐defined DEGs. The 10‐nucleus rule excludes degenerate profiles rather than serving as the stringency filter: it excluded no cell‐type‐level profile and one subcluster‐level profile (SC1 in one POSA animal, two nuclei), and because the next‐smallest profile in the dataset contained 89 nuclei, results are unchanged for any threshold between 10 and 89 nuclei. We confirmed that the cell‐type‐level results remain robust at minimum thresholds of 50, 100 or 200 nuclei (Table S1); at the subcluster level, thresholds above 89 nuclei reduce individual populations to two replicates per group and the additional genes so recovered reflect small‐sample instability rather than increased sensitivity.

Functional enrichment analysis was performed separately for upregulated and downregulated gene sets within each cell type using the STRING database (v12.0) (Szklarczyk et al. 2023). Gene symbols were mapped to STRING identifiers using the STRING API, and enrichment was assessed against curated functional annotations including Gene Ontology biological processes, KEGG pathways, and Reactome pathways. Enrichment results were filtered using a false discovery rate (FDR) threshold of 0.05. Enrichment analyses were performed independently for each cell type to preserve cell type–specific biological signal using STRING's internal background gene universe for each cell type.

### 2.7 Subclustering of the Oligodendrocyte Lineage and Microglia

To resolve cell states within lineages, the oligodendrocyte lineage (ODC1, ODC2, OPC) and microglia (MGL1 to MGL3) were subclustered independently. For each lineage, raw counts for the constituent nuclei were extracted, normalized to 10,000 counts per nucleus and log‐transformed, and highly variable genes were selected within each animal; ribosomal protein and mitochondrial genes were excluded from the feature set so that they could not drive the embedding. The top 30 principal components were integrated across the six animals with Harmony (Korsunsky et al. 2019), and nuclei were clustered with the Leiden algorithm (resolution 0.4, reported; resolutions 0.2 to 1.0 were inspected). Subcluster marker genes were identified with Wilcoxon rank‐sum tests, and cell‐state identities were assigned using published signature panels for oligodendrocyte maturation states, disease‐associated and interferon‐response oligodendrocytes, homeostatic and disease‐associated microglia, interferon‐response microglia, border‐associated macrophages, and proliferation. Cross‐lineage signature scores (neuronal, oligodendrocyte, astrocytic, microglial and vascular) were computed for every subcluster so that populations defined by ambient RNA or doublets could be identified as such rather than interpreted as biological states.

### 2.8 Gene Regulatory Network and Regulon Activity Analysis

To infer cell type–specific gene regulatory programs and transcription factor activity, we applied the SCENIC framework using pySCENIC (v0.12.1) (Van de Sande et al. 2020). Analyses were performed on the filtered single‐nucleus RNA‐seq dataset using expression matrices exported in loom format. The SCENIC workflow was executed in three stages: gene regulatory network inference, regulon definition via motif enrichment, and regulon activity scoring.

In the first stage, gene regulatory networks were inferred using GRNBoost2 (Moerman et al. 2019), which identifies putative transcription factor–target gene relationships based on expression co‐variation. Only transcription factors listed in a curated mouse transcription factor database were considered as candidate regulators. Network inference was parallelized using multiple workers to accommodate the large dataset.

In the second stage, putative regulatory modules were refined by cis‐regulatory motif enrichment analysis using RcisTarget databases for the mouse genome (mm10). Motif enrichment was performed using both proximal (±500 bp) and distal (±10 kb) regulatory region databases, and regulons were defined as transcription factors with statistically enriched motif‐supported target gene sets. Dropout masking was enabled during motif enrichment to improve robustness in sparse single‐nucleus data.

In the final stage, regulon activity was quantified at the single‐nucleus level using AUCell, which computes the enrichment of regulon target genes within the ranked expression profile of each nucleus. Regulon activity scores were stored as a separate assay and used for downstream analyses.

To identify cell type–specific regulatory programs, regulon specificity scores (RSS) were computed for each regulon across annotated cell type clusters. RSS was calculated based on the Jensen–Shannon divergence between the distribution of regulon activity across all nuclei and the indicator distribution of each cell type, yielding values between 0 and 1, with higher values indicating greater cell type specificity. Regulons with high RSS values were considered selectively active within specific cell populations.

Differential regulon activity between POSA and control conditions was assessed within each cell type cluster using an approach analogous to differential gene expression analysis. Regulons were considered differentially active if they met both statistical and effect size criteria (adjusted *p* value < 0.05 and absolute log2 fold change in AUCell score > 0.25). Differentially active regulons were analyzed separately by direction of change and integrated with transcription factor expression and target gene expression for downstream visualization and interpretation.

### 2.9 Comparison of snRNA‐Seq Data With Published RNA‐Seq and Proteomics Datasets

Lists of cell type‐ and subcompartment‐specific proteins were obtained from published datasets: human myelin‐axon interface (Cai et al. 2025), astrocyte leaflet (Soto et al. 2023), and astrocyte endfoot (Hill et al. 2025). Lists of proteins were compared with lists of genes differentially expressed in POSA within oligodendrocytes or astrocytes to identify overlap. The BrainRNAseq dataset (Cahoy et al. 2008) was used for comparisons of normal gene expression between genes within oligodendrocytes or across cell types for *Slc2a1*.

### 2.10 Histology

P70 mice were anesthetized with isoflurane and perfused with phosphate buffered saline (PBS) followed by 4% paraformaldehyde. Brains were harvested, cryoprotected overnight in 20% sucrose, and then embedded for cryostat sectioning. For immunostaining, free‐floating sections were washed three times in PBS, blocked with 10% donkey serum in PBS with 0.3% Triton for 60 min, and incubated with primary antibodies in blocking solutions overnight at 4°C. The next day, sections were washed in PBS with 0.3% Triton, incubated with secondary antibodies for 60 min, stained with DAPI or Hoechst, and washed three times with PBS prior to mounting. Primary antibodies were rabbit anti‐Glut1 (abcam, ab115730, 1:500), goat anti‐Iba1 (Abcam, ab5706, 1:500), rabbit anti‐Lrrk2 (Invitrogen, PA1‐16990, 1:500), rabbit anti‐myelin basic protein (MBP; Atlas Antibodies, HPA049222, 1:2500), rabbit anti‐Ndufs4 (Invitrogen, PA5‐21667, 1:500), rabbit anti‐Olig2 (EMD‐Millipore, AB9610, 1:500), rabbit anti‐QDPR (Proteintech, 14908‐1‐AP, 1:200), rabbit anti‐Sox2 (Millipore, AB5603, 1:500), mouse anti‐Sox8 (Santa Cruz Biotechnologies, sc‐374445, 1:500), and rabbit anti‐Tfe3 (Proteintech, 14480‐1‐AP, 1:250). Secondary antibodies (1:1000, Invitrogen) were produced in donkey against target species and conjugated with Alexa Fluor 488, 594, or 647 fluorophores.

### 2.11 Image Acquisition and Analysis

Fluorescent images were acquired using a confocal microscope (Leica, SP8X) with 10, 20, and 63× oil objectives. Cell counts were obtained from Z stack images throughout the depth of the section. For quantification of fluorescence intensity, all samples were stained in parallel, images were acquired in one session on the same day, and identical acquisition settings (laser power, detector gain, and offset settings) were maintained for all samples. To prevent spectral bleed‐through between fluorophores, images were acquired using sequential scanning mode. The pinhole was set to 1 Airy Unit for the longest wavelength and maintained consistently across all channels to ensure uniform optical sectioning. To ensure representative analysis and mitigate spatial selection bias, a systematic random sampling strategy was employed. For each animal, 3 images were acquired from separate sections for each protein examined. Areas exhibiting tissue folds or edge artifacts were excluded from the sampling pool. Images were acquired from the dentate gyrus stratum moleculare and the corpus callosum superficial to the dorsal hippocampus. Single channel intensity was measured in regions of interest using FIJI (ImageJ version 1.54f). Intensity values were corrected by subtracting local background measured from adjacent non‐expressing regions to account for localized tissue autofluorescence and nonspecific antibody binding. Cell counts were obtained by counting cells labeled with cell‐type‐specific antibodies or by reporter mice. Counts were performed using Z‐stack images captured throughout the entire depth of the section using the optical disector method. When measuring fluorescence intensity in cell types of interest, regions of interest were defined by obtaining masks from cell‐type labels (e.g., Iba1, Olig2) obtained in a separate channel and corrected intensity was measured within masked regions separately for each cell with the measure function. Experimenters were blinded to sample identity during image acquisition and analysis to ensure rigor and reproducibility.

Astrocyte and oligodendrocyte morphology was examined using Imaris (v. 10) (Cheng et al. 2023). Individual cells were reconstructed using the filament tool, applying identical settings for processing each sample. Morphological measures such as Sholl intersections and process length were automatically quantified from reconstructions. Because tamoxifen was administered at P12–16, the NG2‐CreERT driver permanently labeled the pool of OPCs present at that developmental window. By the time of assessment at P70, the tdTomato+ population consists of a mix of both OPCs (which self‐renewed or remained as precursors) and mature, myelinating oligodendrocytes that differentiated from the originally labeled OPC pool during the intervening weeks. In our morphological analysis, we omitted examination of cells with bipolar morphology and ovoid cell bodies, characteristic of OPCs. Notably, the majority of tdTomato+ cells at P70 had multipolar, complex morphology and rounded cell bodies, characteristic of mature oligodendrocytes. Microglia morphology was quantified using a previously characterized automated pipeline for examining microglia morphological states, MicrogliaMorphology, as previously described (Kim et al. 2024).

A random forest machine‐learning model was trained to classify individually reconstructed microglia as healthy or POSA using the scikit‐learn library in Python. The model was trained using the RandomForestClassifier class with 100 estimators, a random state of 0, and a maximum depth of 3. The random forest model was trained to analyze feature importances in reconstructed microglia images by processing the cell‐wise MicrogliaMorphology quantifications in tabular format and classifying the cells as healthy or POSA. Training and validation included 5‐fold cross‐validation. During training, the model learns the importance of individual morphological features in numerical form for classification and represents the importances as Gini scores. Complementarily, we calculated SHapley Additive exPlanations (SHAP) scores for every raw feature value to showcase how the model internally ranks the importance of microglia morphology features, and their raw values, for classifying microglia as healthy or POSA.

### 2.12 Western Blot

Flash frozen hippocampus was homogenized in RIPA buffer. Lysates were centrifuged for clarification and protein concentration was determined by Bradford Assay. Equal amounts of protein were separated by Mini‐PROTEAN TGX 4%–15% Gradient Precast Gels for Lrrk2 (Bio‐Rad, Cat #4561083) or handcast gels and transferred to nitrocellulose membranes using the Bio‐rad Mini Turbo Transfer machine. Membranes were blocked in Bio‐Rad Every Blot Blocking Buffer and incubated with primary antibodies at 4′C, including anti beta‐actin (1:3000; GeneTex, GTX637675, Rabbit), anti‐LRRK2 (1:500, Abcam, ab133474, Rabbit), anti‐SOX8 (1:1000; Santa Cruz Biotechnologies, sc‐374445, Mouse), anti‐QDPR (1:1000; Proteintech, 14908‐1‐AP, Rabbit), anti‐TFE3 (1:500; Proteintech, 14480‐1‐AP, Rabbit), anti‐GLUT1 (1:500, abcam, ab115730, Rabbit), anti‐GLUT3 (1:1000, abcam, ab315169, Rabbit). The following day, membranes were washed with TBST and incubated with horseradish peroxidase‐conjugated secondary antibodies (rabbit or mouse, 1:5000) for 1 h at room temperature. Signals were detected with Western Bright ECL Spray (Advansta, Cat #K‐12049‐D50) and imaged using Bio‐Rad ChemiDoc MP Imaging System. Images were analyzed using the ImageJ Gel Analyzer plugin.

### 2.13 Quantitative Real‐Time PCR

RNA was extracted from dissected hippocampi using the Qiagen RNeasy kit according to manufacturer's instructions. cDNA was synthesized using the iScript gDNA Clear cDNA Synthesis Kit (Bio‐Rad, 1725034) according to manufacturer's instructions. Real‐time qPCR was performed using SYBR Green (Thermo Fisher, 4309155) on a Bio‐Rad CFX96 Real‐Time PCR Detection System. Primer sequences are listed in Table S2.

### 2.14 Statistics

Sample sizes and statistical tests are noted in figure legends. Sample sizes were based on past work using similar methods. Mice were randomly allocated to experimental groups. All analyses were done by experimenters blind to group allocation. Data variance was compared between groups using *F* tests where appropriate. Unpaired *t*‐tests were used for group comparisons where variance was not different between groups. Welch's corrected *t*‐tests were used for group comparisons where variance was significantly different between groups. Nested *t*‐tests were used when examining multiple cells or regions of interest per animal per group. Sholl analysis data were analyzed with two‐way repeated measures analysis of variance (ANOVA). Data are presented using mean ± standard error of the mean (SEM) unless noted otherwise.

## 3 Results

### 3.1 A Mouse Model of POSA Featuring Learning and Memory Deficits

We recently developed a preclinical mouse model of POSA based on human polysomnographic data showing that oxygen saturation and duration of hypoxia are key drivers of cognitive deficits in POSA (Chandrakantan et al. 2020, 2025). This model uses forced intermittent hypoxia during sleep hours to recapitulate hypoxic episodes seen in human patients. We previously reported that this model involves deficits on novel place recognition and novel object recognition memory tasks that rely on perirhinal cortex and hippocampal circuits (Chandrakantan et al. 2025). These deficits in recognition learning and memory reflect translational relevance to the human condition. To further characterize the extent of deficits in this model, we tested mice on a within‐subjects pattern separation task with low stimulus contrasts that is highly sensitive to dentate gyrus‐dependent learning and memory function (van Goethem et al. 2018). The pattern separation task involves multiple test sessions with varying degrees of difficulty in distinguishing object location and type. This task involves two place discrimination tests of varying difficulty and an object type discrimination test (Figure 1A–C and Methods). We exposed mice to intermittent hypoxia (POSA) or normoxia (control) from P14 to P70, targeting a comparable development window to human children with POSA, and then began the four‐day pattern separation task at P70 (Figure 1A–C). POSA mice demonstrated significantly impaired ability in pattern separation ability across test trials, confirming learning and memory deficits in this model (Figure 1C,D). Thus, this mouse model is suitable for examining the cellular and molecular underpinnings of learning and memory deficits in POSA.

### 3.2 Single Nucleus RNA Sequencing of the Hippocampus in POSA

**FIGURE 2:**
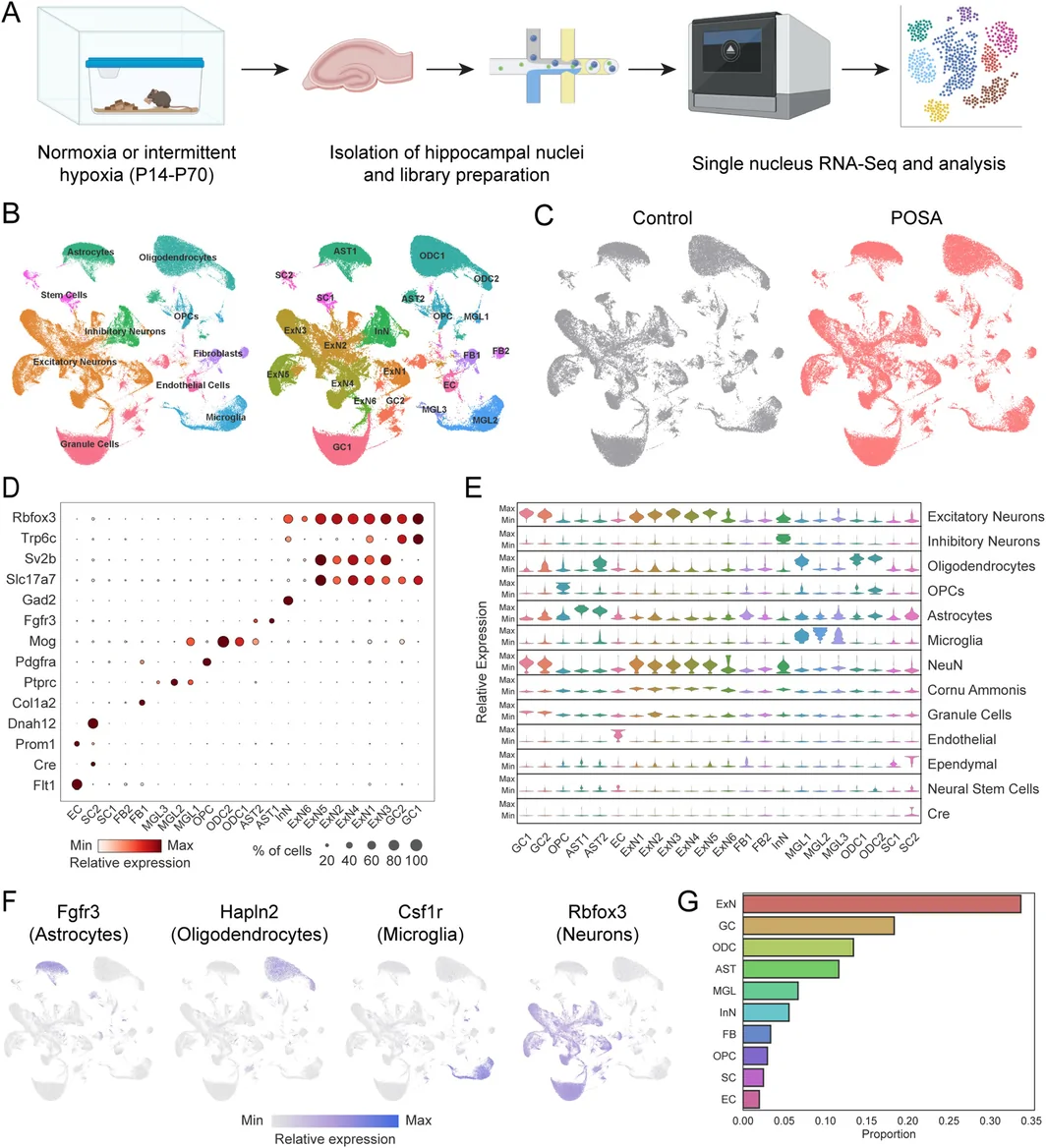
Single nuclei RNA‐sequencing of the hippocampus in POSA. (A) Experimental pipeline for snRNA‐seq of hippocampi from control (*n* = 3) and POSA (*n* = 3) mice. (B) UMAP (uniform manifold approximation and projection) plots showing cell types (left) and subclusters (right) in the merged snRNA‐seq dataset. Abbreviations are defined in Table S3. (C) The UMAP embedding from (B) shown separately by experimental group, with control nuclei in gray and POSA nuclei in red. All annotated subclusters are represented in both groups; differences in relative abundance are evaluated separately. (D) Heatmap of selected marker gene expression by subcluster. (E) Violin plots illustrating gene module expression across subclusters and cell types. (F) Feature plots illustrating marker gene expression selectivity. (G) Proportions of cell types in the merged snRNA‐seq dataset. Cell‐type abbreviations: AST, astrocytes; EC, endothelial cells; ExN, excitatory neurons; FB, fibroblasts; GC, granule cells; InN, inhibitory neurons; MGL, microglia; ODC, oligodendrocytes; OPC, oligodendrocyte precursor cells; SC, neural stem cells.

To define cell type‐specific gene expression changes that may be involved in cognitive deficits in POSA we performed single nucleus RNA sequencing (snRNA‐seq) of the hippocampus of mice at P70 following POSA or control manipulations from P14 to P70 (Figure 2A). We focused on the hippocampus because learning and memory impairments often occur in human POSA patients and we observe correlated impairments in our mouse model. In addition, we previously observed deficits in postnatal neurogenesis in this POSA model (Chandrakantan et al. 2025). We used Nestin‐CreER mice for this experiment to facilitate computational analysis of neural stem cells identified by RNA expression of Cre recombinase. After removing low quality nuclei, we generated a transcriptome dataset with 70,586 control nuclei and 83,121 POSA nuclei. Clustering revealed the presence of 10 broad cell type clusters, which could be further divided into 22 subclusters based on selective expression of marker genes (Figure 2B–G, Figures S1 and S2, and Table S3). Differential expression was assessed within each subcluster; Figure S3 summarizes DEG overlap across broad cell types. The largest numbers of cell‐level MAST‐defined differentially expressed genes were found in the MGL2 microglia subcluster (1266 genes) and the ODC2 oligodendrocyte subcluster (503), the GC1 granule‐cell subcluster (479), the ExN2 excitatory‐neuron subcluster (427), and the SC1 neural stem cell subcluster (305). Endothelial cells, fibroblasts, and oligodendrocyte precursor cells showed the fewest changes (4–57 genes). Gene expression changes in oligodendrocytes, microglia, and neural stem cells implicate altered myelination, immune homeostasis, and neurogenesis, respectively—processes that could collectively contribute to the learning and memory deficits observed in POSA. The changes in granule‐cell and excitatory‐neuron subclusters suggest potential disruption of hippocampal circuit function, which may arise either through direct effects of POSA on neurons (Figure S2) or secondarily to alterations in glial cell function. Overall, this initial analysis suggests that multicellular gene expression changes may be involved in cognitive deficits in POSA. We therefore sought to examine cell type‐specific molecular and cellular changes within the hippocampus in POSA, focusing on non‐neuronal cell types.

We note that the number of differentially expressed genes per subcluster reflects statistical power as well as the magnitude of the underlying response: across subclusters it scales with both the number of nuclei (Spearman rho = 0.69, *p* = 4 × 10^−4^) and the median number of genes detected per nucleus (rho = 0.56, *p* = 0.007). These counts should therefore be read as indicating where transcriptional change is detected most sensitively, rather than as a ranking of effect size.

To evaluate whether the cell‐level differential‐expression estimates were preserved when the biological replicate was used as the analysis unit, we performed pseudobulk analyses with edgeR‐QLF and DESeq2 (Figure S4 and Table S4). Across assessable genes, MAST and pseudobulk log2 fold changes were strongly correlated (Spearman *ρ* = 0.87 for edgeR and 0.83 for DESeq2). Among MAST‐defined DEGs, 99.7% and 99.4%, respectively, showed the same direction of change, with fold‐change correlations of 0.95 and 0.94. However, only 0.5% and 2.2%, respectively, also met pseudobulk FDR < 0.10. Thus, pseudobulk analysis supported the direction and relative magnitude of the cell‐level effects but yielded sample‐level gene‐wise significance for a smaller subset under the more stringent replicate‐level analysis with three biological replicates per group. We therefore treated the MAST DEG lists as discovery‐level and report pseudobulk‐significant genes separately. This analysis guided subsequent in vivo examination glial dysfunction in POSA.

### 3.3 Loss of Hippocampal Neural Stem Cells in POSA

**FIGURE 3:**
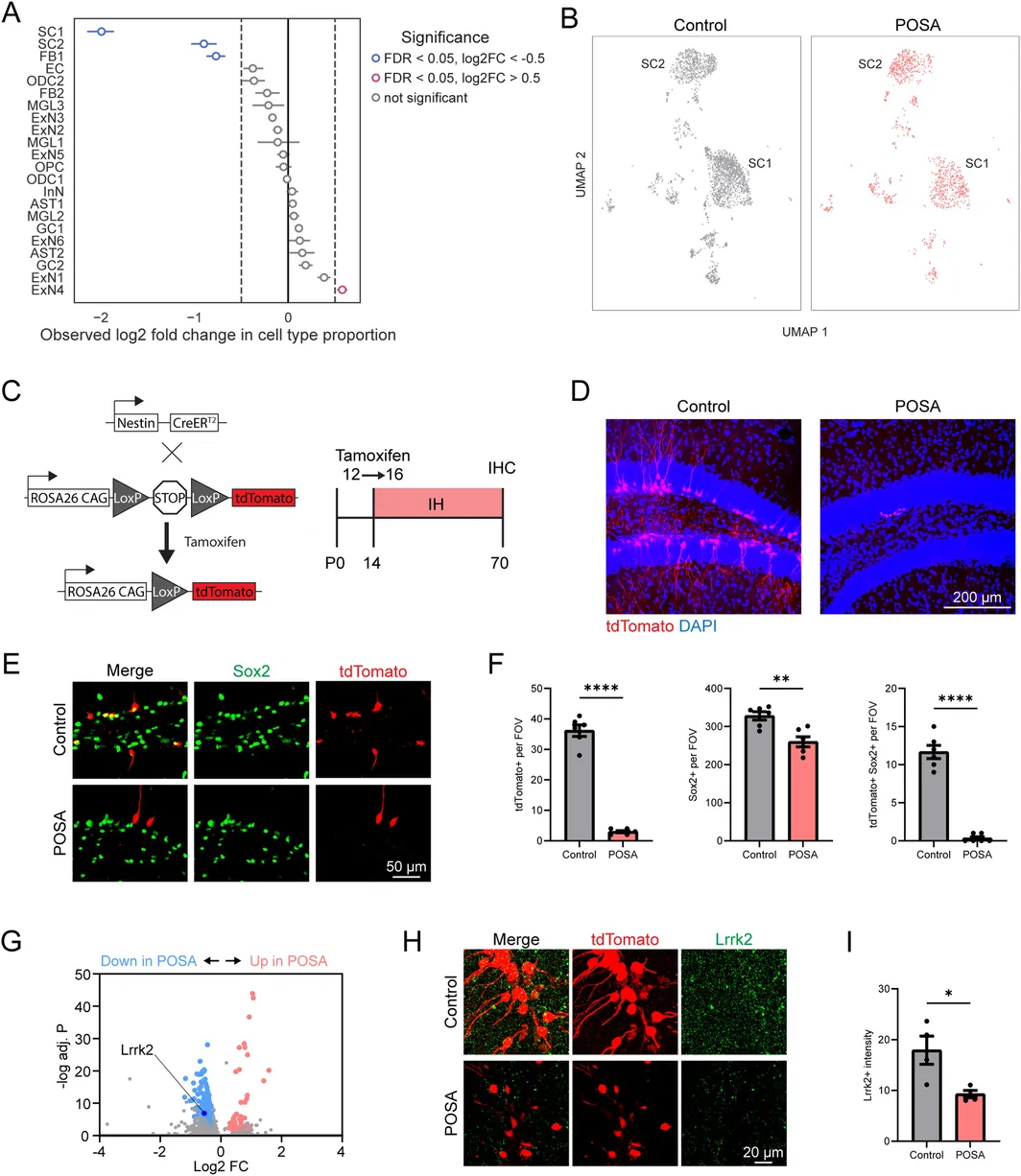
Loss of neural stem cells in POSA. (A) Change in snRNA‐seq cell type proportions in POSA relative to control samples. SC1 and SC2 stem cell subclusters were significantly diminished in POSA. (B) UMAP plots illustrating neural stem cells identified in control and POSA snRNA‐seq samples. About 2673 stem cells were identified in control samples, 1119 stem cells were identified in POSA samples. (C) Schematic of transgenic approach for tamoxifen‐inducible labeling of neural stem cells and experimental timeline. IH, intermittent hypoxia. IHC, immunohistochemistry. (D) Representative widefield images of tdTomato+ neural stem cells and progeny at P70 in the subgranular zone and dentate gyrus. (E) Representative confocal images of Sox2+ tdTomato+ neural stem cells in the subgranular zone. (F) Quantification of tdTomato+, Sox2+, and tdTomato+Sox2+ cells within the subgranular zone. **p = 0.0027, ****p < 0.0001 unpaired two‐tailed *t*‐tests, *n* = 6 mice per group. (G) Volcano plot of differential gene expression in POSA relative to control. *Lrrk2* expression was significantly reduced in POSA. (H) Representative confocal images of LRRK2 protein expression within the subgranular zone. (I) Quantification of LRRK2+ intensity. *p = 0.048, Welch's corrected *t*‐test, *n* = 4 mice per group.

Examination of the proportions of transcriptomically identified cell types from our snRNA‐seq dataset revealed a significant loss of neural stem cells in POSA mice (SC1 and SC2 subclusters) (Figure 3A,B). Notably, the lack of difference in mature granule cell numbers is consistent with our previous findings examining NeuN+ cells within the dentate gyrus (Chandrakantan et al. 2025). To validate the loss of neural stem cells in vivo, we generated Nestin‐CreER; Rosa‐CAG‐LSL‐tdTomato mice, which enable tamoxifen‐inducible expression of tdTomato in neural stem cells and their progeny (Williamson et al. 2023). We injected mice with tamoxifen from P12–P16 to induce tdTomato expression and subjected mice to control or intermittent hypoxia conditions from P14–P70. We harvested brains at P70 to examine the number of neural stem cells within the subgranular zone, a postnatal neurogenic niche within the hippocampus. Using Sox2 as a neural precursor cell marker (Williamson et al. 2023), we observed significantly fewer tdTomato+, Sox2+, and tdTomato+ Sox2+ cells within the subgranular zone in POSA mice. These observations corroborate the snRNA‐seq data and indicate that POSA causes a substantial loss of neural stem cells and their progeny. These findings are consistent with our previous observations of impaired neurogenesis in this POSA model (Chandrakantan et al. 2025) and highlight neural stem cell loss as a potential contributing factor to impairments in learning and memory.

Next, we sought to identify potential gene expression changes that could underlie the loss of neural stem cells and impairments in neurogenesis in POSA. LRRK2 (leucine‐rich repeat kinase 2) is a protein with kinase and GTPase domains that functions both in a cell‐autonomous and secreted manner. LRRK2 has been previously reported to regulate proliferation of neural precursor cells and morphogenesis of postnatally born neurons in the subgranular zone (Winner et al. 2011; Berwick and Harvey 2013). We found that *Lrrk2* RNA expression was significantly reduced in neural stem cells in POSA samples (Figure 3G). Concordantly, *Lrrk2* RNA expression was also significantly reduced in bulk hippocampal tissue from POSA mice harvested at P70 and assessed by qPCR (Figure S5). Next, we examined protein level expression of LRRK2 within the subgranular zone at P70 by immunohistochemistry. We observed a significant reduction in LRRK2+ intensity, consistent with a loss of both intracellular and secreted LRRK2 (Figure 3H,I). We confirmed the loss of LRRK2 protein by western blot on hippocampal lysate collected at P70 (Figure S6). Thus, reduced Lrrk2 expression is a candidate mediator of neurogenesis impairments in POSA.

We also identified transcriptional changes in neural stem cells related to the function of mitochondrial complex I, which is essential for neural stem cell proliferation and differentiation, and morphogenesis of new neurons (Bolea et al. 2019; Cabello‐Rivera et al. 2019; Inak et al. 2021). In particular, nicotinamide adenine dinucleotide hydride (NADH) dehydrogenase ubiquinone iron–sulfur protein 4 (*Ndufs4*; an essential component of mitochondrial complex I) RNA expression was significantly reduced in neural stem cells in POSA mice. Furthermore, we validated reduced expression of *Ndufs4* RNA with qPCR (Figure S5) and observed reduced NDUFS4 protein within subgranular zone neural stem cells in POSA by immunostaining (Figure S7). Impaired mitochondrial respiratory chain function is another candidate mechanism for stem cell loss and impaired neurogenesis in POSA.

### 3.4 Molecular and Cellular Dysfunction of Oligodendrocytes in POSA

**FIGURE 4:**
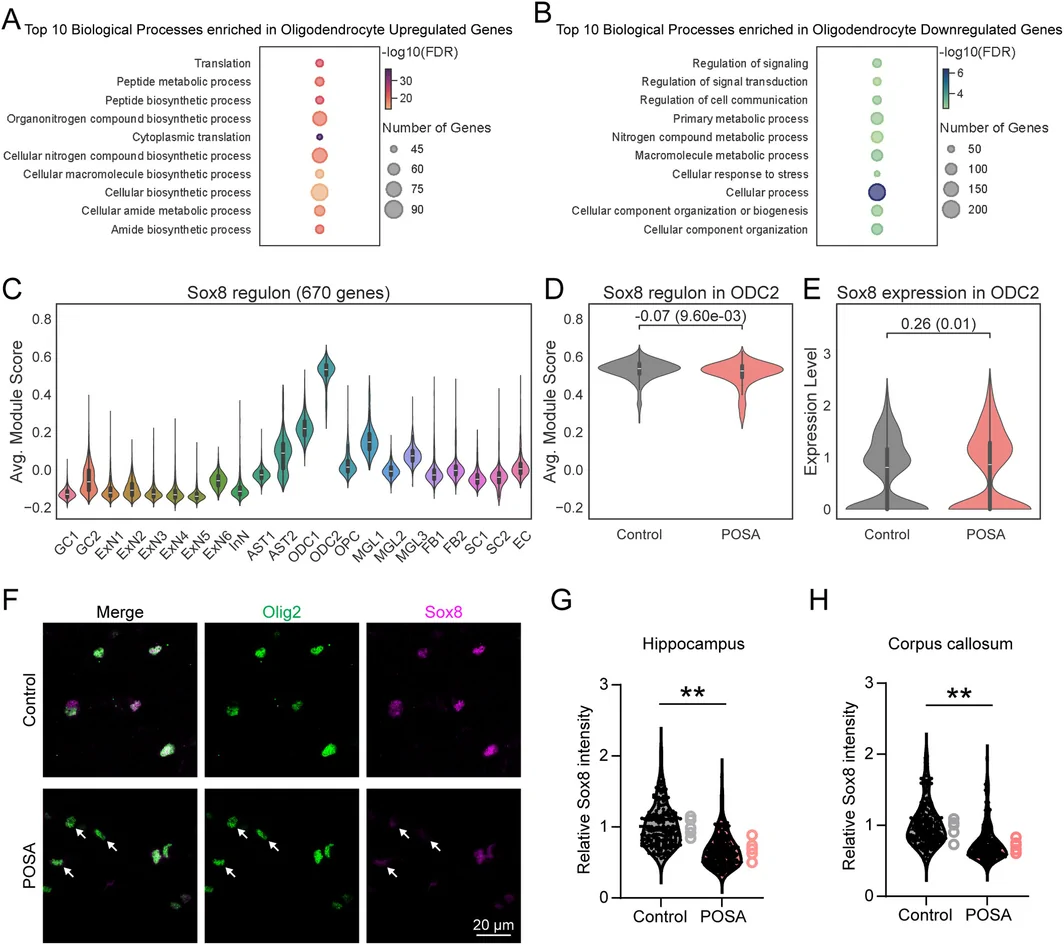
Transcriptional dysregulation of oligodendrocytes in POSA. (A) Gene ontology analysis of genes upregulated in oligodendrocytes in POSA. (B) Gene ontology analysis of genes downregulated in oligodendrocytes in POSA. (C) SCENIC analysis reveals enrichment of a SoxE (Sox8/Sox10) regulon in oligodendrocyte subclusters, particularly ODC2. (D) Per‐nucleus SoxE regulon activity score in the ODC2 subcluster by group. The score was modestly lower in POSA (log2 fold change = −0.07, adjusted *p* = 0.0096). (E) Sox8 RNA expression was modestly but significantly increased in ODC2 in POSA (log2 fold change = 0.26, adjusted *p* = 0.01). (F) Representative confocal images of immunostaining for SOX8 and the oligodendrocyte marker OLIG2. Arrows indicate OLIG2+ cells with low SOX8 expression. (G) Quantification of SOX8+ intensity within OLIG2+ cells in the hippocampal gray matter (dentate gyrus stratum moleculare). **p = 0.0063, nested *t*‐test, *n* = 5 mice per group (rings), *n* = 479 total OLIG2+ cells (dots). (H) Quantification of SOX8+ intensity within OLIG2+ cells in the corpus callosum overlying the hippocampus. **p = 0.0099, nested *t*‐test, *n* = 5 mice per group (rings), *n* = 506 total Olig2+ cells (dots).

In continuing our analysis of the snRNA‐seq dataset, we next examined oligodendrocytes, which exhibited hundreds of differentially expressed genes between control and POSA groups (Figure S3). Gene ontology analysis revealed enriched terms related to cellular metabolism, communication, and organization (Figure 4A,B). These processes are putatively important for oligodendrocyte function pertaining to myelin production and deposition, and establishment of appropriate contact with axons to be myelinated. To define the oligodendrocyte states underlying these changes, we subclustered the oligodendrocyte lineage (ODC1, ODC2, and OPC; 25,115 nuclei) independently of the remaining cell types (Figure S8). This resolved the expected maturation series, comprising oligodendrocyte precursor cells, a proliferating precursor state (*Mki67*, *Top2a*), a differentiation‐committed and newly forming state (*Bmp4*, *Prom1*, *Enpp6*), and mature myelinating states. ODC2 was recovered as a single discrete subcluster (2186 of 2257 ODC2 nuclei), corresponding to the terminally mature, high‐output myelinating end of the lineage: it showed the highest mature‐oligodendrocyte, MOL5/6‐like (*Klk6*, *Il33*, *Apod*, *Ptgds*) and myelin‐biosynthesis module scores of any subcluster and, beyond *Mog*, was defined by *Cryab*, *Qdpr*, *Ppp1r14a*, *Nkx6‐2*, *Ermn*, *Trf*, *Gjb1*, and *Tmem88b*. Superimposed on this myelin program was a proteostatic signature (*Cryab*, *Hspa1b*, *Ubb*) together with the highest hypoxia‐response score in the lineage, identifying ODC2 as the most biosynthetically committed and correspondingly stressed oligodendrocyte state. Consistent with the power considerations noted above, ODC2 nuclei carry approximately 4.5‐fold the transcript content of ODC1 nuclei (median 5596 versus 1232 UMIs), so ODC2 is the oligodendrocyte subcluster in which transcriptional change is detected most sensitively rather than necessarily the one in which it is largest.

To better understand the gene regulatory mechanisms involved in oligodendrocytic transcriptional alterations in POSA we applied single‐cell regulatory network inference and clustering (SCENIC) analysis (Aibar et al. 2017; Van de Sande et al. 2020). SCENIC identified a SoxE (Sox8/Sox10) transcription factor regulon with high activity in oligodendrocytes, particularly within the ODC2 subcluster (Figure 4C). This across‐cell‐type comparison is distinct from the within‐ODC2 POSA‐versus‐control comparison in Figure 4D, in which the SoxE regulon activity score was modestly but significantly lower in POSA (log2 fold change = −0.07, adjusted *p* = 0.0096). Because this change did not meet the prespecified effect‐size threshold for differential regulon activity (|log2 fold change| > 0.25), we interpret it as a hypothesis‐generating signal and examine SOX8 protein expression below. We note that *Sox8* and *Sox10* are SoxE‐family paralogues with near‐identical binding motifs, and that SCENIC cannot resolve them: the same motif metacluster provides the top‐scoring evidence for both regulons, they share half of their target genes, and their per‐nucleus activity scores are essentially collinear (Pearson *r* = 0.985), with the *Sox10* regulon showing an equivalent reduction in ODC2 in POSA (Figure S9). Notably, SOX8 is required for myelin production and maintenance in oligodendrocytes (Turnescu et al. 2018). *Sox8* RNA expression was decreased in POSA bulk hippocampus qPCR (Figure S5). Interestingly, *Sox8* expression was modestly but significantly increased in the ODC2 subcluster in POSA in our snRNA‐seq dataset (log2 fold change = 0.26, adjusted *p* = 0.01; Figure 4E), despite the lower regulon activity score. To extend these findings, we performed immunohistochemistry for SOX8 with co‐staining for the oligo‐lineage marker OLIG2. We observed significantly reduced expression of SOX8 protein within OLIG2+ cells (Figure 4F–H), consistent with a loss of predicted SoxE regulon activity within oligodendrocytes. Concordantly, western blot showed a trend for reduced SOX8 in bulk hippocampal POSA tissue (Figure S6). These data indicate that SOX8 protein abundance is reduced despite increased *Sox8* transcript abundance in the ODC2 subcluster, suggesting potential dysregulation downstream of transcription that could affect oligodendrocyte function in POSA. Together, these data suggest that reductions in SOX8 transcriptional regulation could affect oligodendrocyte function in POSA.

**FIGURE 5:**
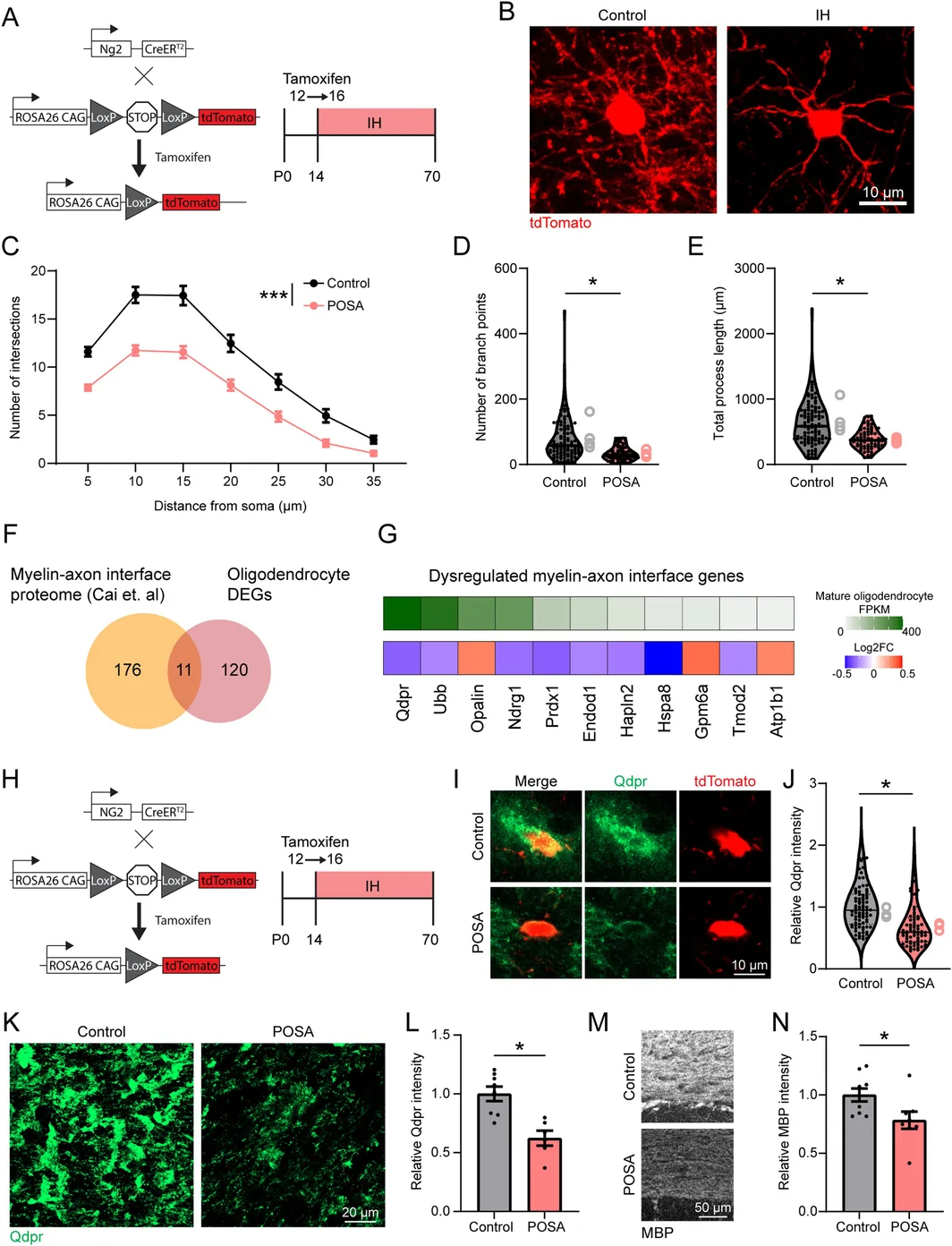
Molecular and cellular dysfunction of oligodendrocytes in POSA. (A) Schematic of transgenic approach and experimental timeline for tamoxifen‐inducible labeling of oligodendrocyte lineage cells. (B) Representative images of tdTomato+ oligodendrocytes in the corpus callosum dorsal to the hippocampus. (C) Sholl analysis of oligodendrocyte morphological complexity. ***p < 0.0001, group main effect, two‐way repeated measures ANOVA. Sample sizes for morphological analyses: *N* = 5 mice per group (rings), *n* = 106 control cells, *n* = 77 POSA cells (dots). (D) Quantification of branch points per oligodendrocyte. *p = 0.0309, nested *t*‐test. (E) Quantification of total branch length per oligodendrocyte. *p = 0.0199, nested *t*‐test. (F) Comparison and overlap between human myelin‐axon interface proteins (Cai et al. 2025) and differentially expressed genes (DEGs) in oligodendrocytes. (G) Heatmap showing gene expression (Fragments Per Kilobase of transcript per Million mapped reads, FPKM) in healthy mature oligodendrocytes (from BrainRNAseq database) and change (Log2FC) in POSA of the 11 overlapping genes from Panel F. (H) Schematic of transgenic approach and experimental timeline for tamoxifen‐inducible labeling of oligodendrocyte lineage cells. (I) Representative confocal images of QDPR protein expression in tdTomato+ oligodendrocytes within the hippocampal gray matter (dentate gyrus stratum moleculare). (J) Quantification of QDPR protein expression in the hippocampus. *p = 0.0471, nested *t*‐test, *n* = 3 mice per group (rings), *n* = 86 control cells, *n* = 72 POSA cells (dots). (K) Representative confocal images of QDPR protein expression in the corpus callosum overlying the hippocampus. (L) Quantification of QDPR protein expression in the corpus callosum. *p = 0.0195, nested *t*‐test, *n* = 3 mice per group, *n* = 8 control regions of interest, *n* = 6 POSA regions of interest. (M) Representative images of myelin basic protein (MBP) staining in the corpus callosum. (N) Quantification of MBP+ intensity. *p = 0.0309, unpaired two‐tailed *t*‐test, *n* = 9 control mice, *n* = 8 POSA mice.

Next, we examined cellular aspects of oligodendrocytes to better understand how they are affected by POSA. We generated *Ng2‐CreER; Rosa‐CAG‐LSL‐tdTomato* mice, which enable tamoxifen‐inducible expression of tdTomato in oligodendrocyte progenitors and their mature progeny. We injected mice with tamoxifen from P12–P16 to induce tdTomato expression and subjected mice to control or intermittent hypoxia conditions from P14 to P70 (Figure 5A). We harvested brains at P70 to examine oligodendrocyte morphology within the corpus callosum overlying the hippocampus. A highly branched oligodendrocyte morphology is essential for myelinating nearby axons. We found that the morphological complexity of oligodendrocytes was significantly reduced in POSA mice, as measured with Sholl analysis, number of branch points, and total process length (Figure 5B–E and Figure S10). Thus, oligodendrocytes exhibit aberrant molecular and cellular properties in POSA.

Oligodendrocytes ensheathe axons with myelin to promote conduction of action potentials. The myelin‐axon interface is characterized by a highly structured molecular architecture with a unique proteome (Cai et al. 2025). The myelin‐axon interface proteome is disrupted in several diseases, which contributes to neurological deficits (Cai et al. 2025). To examine whether the myelin‐axon interface might be disrupted in POSA we compared differentially expressed genes in POSA oligodendrocytes to the human myelin‐axon interface proteome. Strikingly, approximately 10% of differentially expressed genes in oligodendrocytes in POSA encoded proteins that are part of the myelin‐axon interface (Figure 5F). Among the dysregulated myelin‐axon interface genes we identified, *Qdpr* (quinoid dihydropteridine reductase) displays the highest expression in healthy mature oligodendrocytes (Cahoy et al. 2008), is specifically expressed in mature oligodendrocytes and myelin (Siems et al. 2025), and was significantly downregulated in POSA (Figure 5G). *Qdpr* RNA abundance was also significantly reduced in POSA samples relative to controls as measured by qPCR (Figure S5). We next examined protein level expression of QDPR via immunostaining. We found significant reductions of QDPR expression within both the hippocampal gray matter and white matter of the overlying corpus callosum (Figure 5H–L). Similarly, western blot showed a trend for decreased QDPR in bulk hippocampal tissue from POSA samples (Figure S6). The loss of QDPR in POSA may contribute to aberrant myelination. To directly examine myelination in this POSA model we measured myelin basic protein (MBP) within the corpus callosum overlying the hippocampus at P70 using immunostaining. We found significantly reduced MBP+ intensity in POSA mice, consistent with impaired myelination (Figure 5M,N). Together, these findings demonstrate that POSA involves molecular and cellular dysfunction of oligodendrocytes and associated myelination deficits, which may contribute to cognitive impairments.

### 3.5 Molecular and Cellular Changes in Microglia in POSA

**FIGURE 6:**
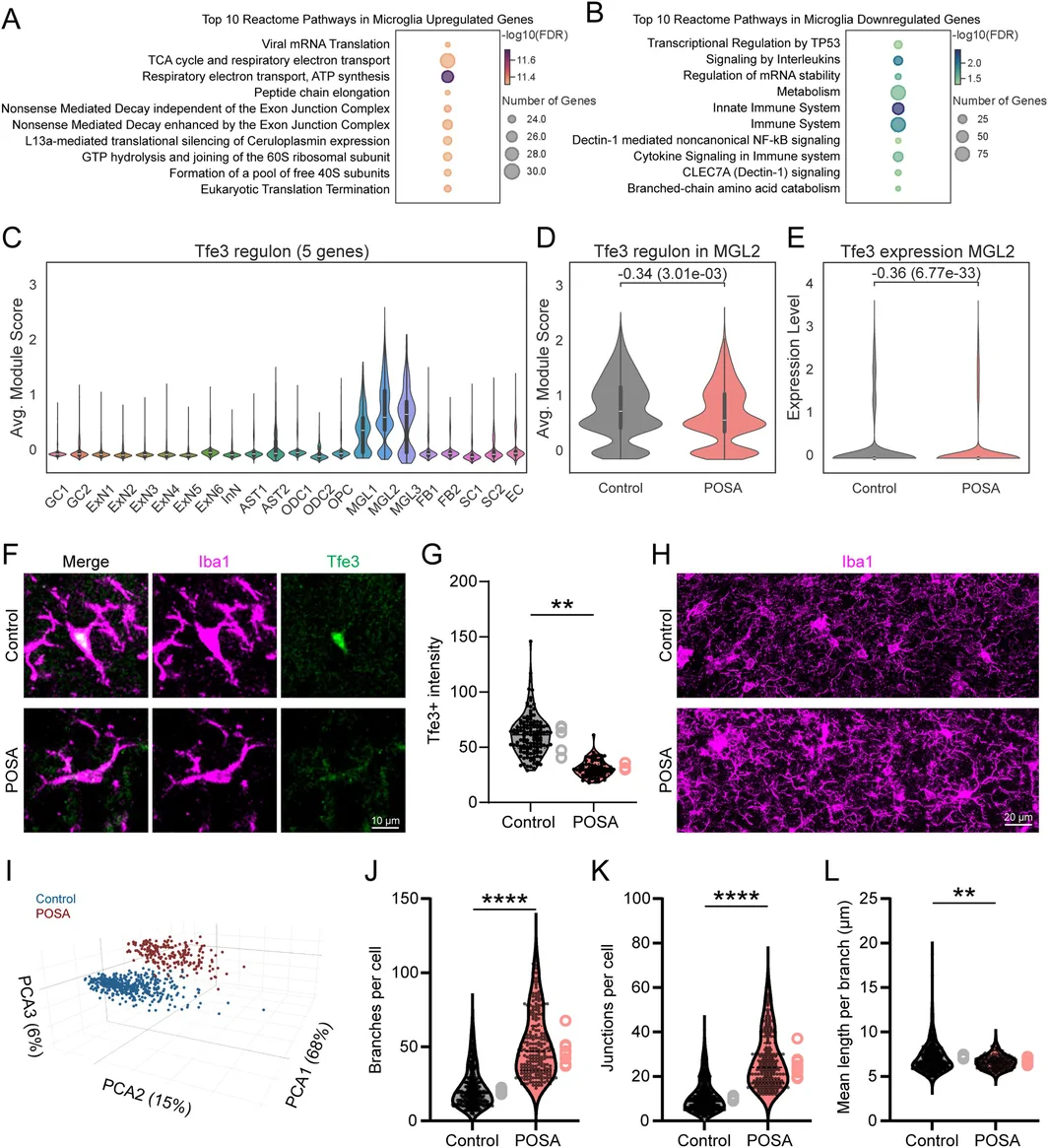
Cellular and molecular alterations of microglia in POSA. (A) Reactome analysis of genes upregulated in microglia in POSA. (B) Reactome analysis of genes downregulated in microglia in POSA. (C) SCENIC analysis reveals enrichment of a Tfe3 regulon in microglia subclusters, particularly MGL2. (D) Per‐nucleus Tfe3 regulon activity score in the MGL2 subcluster by group. The score was reduced in POSA (log2 fold change = −0.34, adjusted *p* = 0.0030). (E) *Tfe3* RNA expression is significantly reduced in MGL2 in POSA samples. (F) Representative confocal images of immunostaining for Tfe3 and the microglial marker Iba1 in the hippocampus. (G) Quantification of TFE3+ intensity within Iba1+ cells. **p = 0.0019, nested *t*‐test, *n* = 5 mice per group (rings), *n* = 129 control cells, *n* = 77 POSA cells (dots). (H) Representative confocal images of Iba1 staining to illustrate microglia morphology in dentate gyrus stratum moleculare. (I) PCA plot of morphological features from individually reconstructed microglia. (J) Quantification of branches per cell. ****p < 0.0001, nested *t*‐test. (K) Quantification of junction points per cell. ****p < 0.0001, nested *t*‐test. (L) Quantification of mean branch length per cell. **p = 0.0037, nested *t*‐test. Sample sizes for morphology measures: *N* = 6 control mice (rings), *n* = 8 POSA mice, *n* = 658 total Iba1+ cells (dots).

We next investigated molecular changes in microglia. Notably, the MGL2 subcluster of microglia exhibited the greatest number of cell‐level MAST‐defined differentially expressed genes in POSA across all subclusters in our snRNA‐seq dataset (Figure S3). Examination of Reactome pathways significantly enriched among differentially expressed microglia genes revealed terms related to immune response and metabolism, suggesting an inflammatory state change in POSA (Figure 6A,B). We conducted SCENIC analysis to better understand changes in the gene regulatory mechanisms within microglia subclusters. This analysis revealed a Tfe3 transcription factor regulon with high activity in microglia (Figure 6C). TFE3 is a transcriptional regulator of microglial development and inflammatory responses (Pastore et al. 2016; Han et al. 2023). In POSA, the activity of the Tfe3 regulon was significantly reduced in the MGL2 subcluster. Concordantly, RNA expression of *Tfe3* was significantly reduced in the MGL2 subcluster, and the combined microglia cluster, in POSA snRNA‐seq samples (Figure 6E), and in bulk POSA tissue measured with qPCR (Figure S5). We next examined TFE3 expression at the protein level in hippocampal microglia at P70 following POSA or control interventions. Consistent with our analysis of snRNA‐seq and qPCR data, we observed significantly reduced TFE3+ protein intensity within Iba1+ microglia. Furthermore, western blot measurement of TFE3 protein also showed a significant reduction in POSA relative to control hippocampal tissue (Figure S6). These findings suggest that intermittent hypoxia in POSA elicits transcriptional changes in microglia in part via reduced TFE3 expression.

Subclustering microglia independently of the remaining cell types (MGL1 to MGL3; 10,255 nuclei) resolved activation states that were not separable at whole‐dataset resolution (Figure S11): a dominant homeostatic population (*P2ry12*, *Tmem119*, *Cx3cr1*, *Selplg*), an interferon‐response population (*Ifit1*, *Ifit3*, *Isg15*, *Mx1*, *Usp18*, *Irf7*), a chemokine‐expressing population (*Ccl3*, *Ccl4*, *Csf1*), border‐associated and perivascular macrophages (*Mrc1*, *Lyve1*, *Pf4*, *Cd163*, *Ms4a7*), and a small population of T and NK cells (*Cd3d*, *Cd3e*, *Nkg7*). We found no evidence for a canonical disease‐associated microglial state, as the *Trem2*‐dependent module (*Cst7*, *Lpl*, *Itgax*, *Clec7a*, *Gpnmb*) was not enriched in any subcluster, indicating that microglial activation in this model is of an interferon and chemokine type. Subclustering further indicated that the smaller MGL1 and MGL3 clusters are dominated by technical rather than biological heterogeneity: MGL1 corresponded almost entirely to nuclei carrying oligodendrocyte transcripts (*St18*, *Nkain2*, *Pde4b*), consistent with microglia‐oligodendrocyte doublets and ambient RNA, and MGL3 partitioned into a ribosome‐high fraction whose abundance tracked sequencing batch, a small choroid plexus fraction (*Ttr*, *Krt8*, *Otx2*), and a high‐mitochondrial low‐quality fraction; both clusters contributed few differentially expressed genes. With three animals per group, no subcluster showed a POSA‐associated difference in abundance that exceeded between‐animal variability, and we therefore report subcluster composition descriptively.

The POSA‐related gene expression changes within microglia suggested a change in inflammatory state. When engaging in an inflammatory response, microglia undergo stereotyped morphological ramification (Kim et al. 2024). We examined microglial morphological states based on Iba1 staining within the hippocampus at P70 using a previously developed pipeline (Kim et al. 2024). Microglia in POSA mice exhibited a starkly more ramified appearance in the dentate gyrus stratum moleculare (Figure 6H). Principle component analysis of nine morphological features derived from reconstructions of individual cells revealed separation of cells from control and POSA mice (Figure 6I and Figure S12). Gini and SHAP scores, calculated from the predictions of a random forest classifier trained to classify cells as healthy or POSA, ranked these features in terms of how important their values are for distinguishing between healthy and POSA cells (Figure S12). Moreover, measures of microglial branching and complexity were significantly increased in POSA samples (Figure 6J–L). Notably, we did not observe ameboid morphology of microglia, suggesting that they assume a low grade or chronic inflammatory state rather than an acute activated state in POSA. Overall, these findings suggest that POSA elicits molecular and cellular changes associated with inflammation in hippocampal microglia.

### 3.6 Molecular and Cellular Dysfunction of Astrocytes in POSA

**FIGURE 7:**
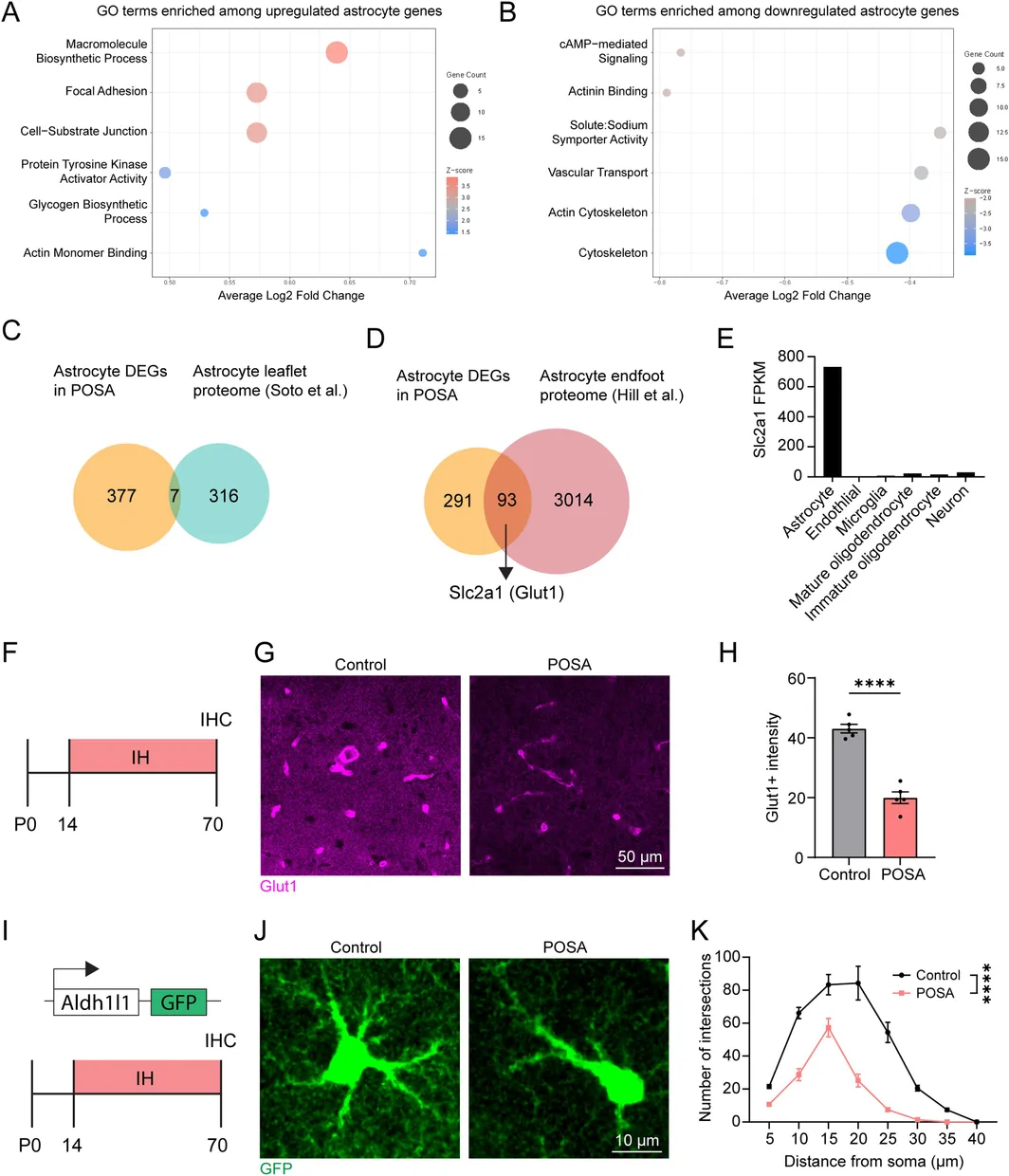
Cellular and molecular dysfunction of astrocytes in POSA. (A) Gene ontology (GO) analysis of genes upregulated in astrocytes in POSA. (B) Gene ontology (GO) analysis of genes downregulated in astrocytes in POSA. (C) Comparison and overlap between astrocyte DEGs and proteins enriched in astrocyte leaflets (Soto et al. 2023). (D) Comparison and overlap between astrocyte DEGs and proteins enriched in astrocyte endfeet (Hill et al. 2025). *Slc2a1* is an endfoot‐enriched protein and its RNA expression is significantly downregulated in POSA astrocytes. (E) Expression of *Slc2a1* across cell types in the healthy adult brain. Data from BrainRNAseq dataset (Cahoy et al. 2008). (F) Experimental timeline. IHC, immunohistochemistry. (G) Representative confocal images of GLUT1 (encoded by *Slc2a1*) protein in the hippocampus. The intense staining along vascular structures is expected given the localization of GLUT1 to astrocytic endfeet. (H) Quantification of GLUT1+ intensity. ****p < 0.0001, unpaired two‐tailed *t*‐test, *n* = 5 mice per group. (I) Schematic of transgenic approach for visualizing astrocyte morphology in *Aldh1l1‐GFP* mice and experimental timeline. (J) Representative confocal images of GFP+ astrocytes in the dentate gyrus stratum moleculare at P70. (K) Sholl analysis of astrocyte morphological complexity. ****p < 0.0001, two‐way repeated measures ANOVA, group main effect, *n* = 10 mice per group.

Astrocytes participate in numerous cell–cell interactions to regulate brain function (Kwon et al. 2025; Lacoste et al. 2025; Williamson, Kwon, et al. 2025). These cell–cell interactions are enabled by a complex morphology that permits direct contact with other brain cell types, especially neurons and blood vessels. Our snRNA‐seq analysis revealed hundreds of differentially expressed genes within astrocytes in POSA. Gene ontology analysis of these differentially expressed genes showed an enrichment for terms related to astrocyte morphology, vascular transport, and metabolism, suggesting that core aspects of astrocyte form and function could be altered in POSA (Figure 7A,B).

Astrocytes possess functionally distinct cellular subcompartments to allow for specialized interactions with synapses and blood vessels (termed leaflets and endfeet, respectively). To better understand whether differentially expressed genes in astrocytes in POSA might relate to either of these subcompartments, we compared the list of genes with lists of proteins that are selectively enriched in astrocyte leaflets (Soto et al. 2023) or endfeet (Hill et al. 2025). This analysis revealed that few (1.8%) differentially expressed genes encoded proteins that localize to leaflets (Figure 7C), whereas approximately 24% of these genes encoded proteins that are enriched within endfeet (Figure 7D). A key function of astrocyte endfeet, which completely ensheathe blood vessels, is to mediate glucose transport from the vasculature into the brain via GLUT1 glucose transporters encoded by *Slc2a1* (Díaz‐Castro et al. 2023; Kwon et al. 2025; Williamson, Murali, and Deneen 2025). Importantly, we found that *Slc2a1* RNA expression was significantly reduced in astrocytes in POSA with both snRNA‐seq and qPCR (Figure 7D and Figure S5). Notably, *Slc2a1* expression is highly specific to astrocytes among brain cell types (Figure 7E). Next, we performed immunostaining and western blot for GLUT1 (encoded by *Slc2a1*) and observed significantly decreased GLUT1 within the hippocampus of POSA mice (Figure 7F–H and Figure S6), in accordance with our snRNA‐seq and qPCR data. Dysfunctional glucose transport due to loss of GLUT1 may contribute to cognitive impairments in POSA.

Next, we asked whether POSA affected astrocyte morphology in the hippocampus. Astrocytes undergo a period of postnatal morphogenesis that is sensitive to experience and neuronal activity (Morel et al. 2014; Cheng et al. 2023; Baldwin et al. 2024; Williamson, Murali, and Deneen 2025) and could conceivably be impacted by POSA. We used *Aldh1l1‐GFP* mice, which express GFP selectively in astrocytes, to visualize the entirety of astrocyte arbors (Cheng et al. 2023). We subjected mice to control or POSA conditions and harvested brains at P70 (Figure 7H). Focusing on the dentate gyrus stratum moleculare, we examined astrocyte morphological complexity using Sholl analysis, as previously described (Huang et al. 2020; Cheng et al. 2023). We found a significant reduction in astrocyte morphological complexity in POSA mice (Figure 7I,J and Figure S10). Together, our findings suggest that molecular and cellular dysfunction of astrocytes—particularly astrocyte endfeet—may be a pathological feature of POSA.

## 4 Discussion

We examined cellular and molecular aspects of pathology in a mouse model of POSA to better understand the nature of cognitive deficits commonly seen in the disease. With snRNA‐seq, we identified broad gene expression changes across cell types within the hippocampus. In particular, we noted substantial transcriptional perturbation within glial cells and validated reduced expression of several genes at the protein level. Using cell type‐specific transgenic labeling approaches, we found that POSA involves a loss of subgranular zone neural stem cells and aberrant morphology of oligodendrocytes, microglia, and astrocytes. Overall, our study suggests that cellular and molecular dysfunction of glial cells may underlie learning and memory deficits in POSA.

Postnatal hippocampal neurogenesis contributes to learning and memory functions, especially discrimination learning and pattern separation (Zhao et al. 2008; Swan et al. 2014; Toda et al. 2019). We found that POSA reduced the number of neural stem cells within the subgranular zone. These findings corroborate and extend our previously reported findings that POSA reduced the number of postnatally born neurons and impaired functional integration of these neurons into the hippocampal circuitry (Chandrakantan et al. 2025). Moreover, reduced numbers of adult‐born neurons were seen in an adult obstructive sleep apnea mouse model (Khuu et al. 2019). Together, these observations indicate that multiple aspects of neurogenesis (stem cell numbers, progeny formation, and neuronal maturation) are impacted in POSA. Leveraging our new snRNA‐seq dataset, we identified and validated reduced expression of LRRK2 and NDUFS4 in neural stem cells in POSA. Given the involvement of both LRRK2 and NDUFS4 in multiple aspects of neurogenesis (Winner et al. 2011; Berwick and Harvey 2013; Cabello‐Rivera et al. 2019), their reductions in POSA are candidate molecular mechanisms for reduced stem cell numbers and aberrant neurogenesis. These findings suggest intermittent hypoxia‐driven neurogenesis impairments as a contributor to cognitive impairments in sleep apnea.

Proper myelination enables diverse cognitive processes, and perturbations of developmental myelination are linked with cognitive impairments (Pan et al. 2020; Khelfaoui et al. 2024). Children undergo extensive myelin development (Bethlehem et al. 2022) that could potentially be impacted by POSA. Prior MRI studies identified altered white matter network topology in children with POSA (Chen et al. 2024). Importantly, these topological changes were related to verbal comprehension impairments. Similarly, aberrant measures of myelin were reported in MRI studies of adults with OSA (Kumar et al. 2014). Here, we observed reduced white matter myelin content in POSA mice. Mechanistically, we identified decreased morphological complexity of oligodendrocytes and reduced protein‐level expression of the myelin transcription factor SOX8 and the myelin‐axon interface component QDPR in POSA. Notably, SOX8 protein was reduced by immunostaining despite a modest increase in *Sox8* mRNA in the ODC2 subcluster. This inverse mRNA‐protein relationship indicates that reduced SOX8 abundance is not explained by reduced transcription and may reflect post‐transcriptional or post‐translational regulation, such as impaired translation or reduced protein stability. The increased transcript abundance could also represent a compensatory response, although this was not directly tested. Because SCENIC infers transcription‐factor activity from the coordinated expression of predicted target genes rather than measuring SOX8 protein directly, the lower regulon activity score is consistent with reduced functional SOX8 output but does not establish the underlying mechanism. Our findings suggest a cellular and molecular basis for aberrant myelination seen in POSA. We further note that the regulon analysis identifies SoxE‐family activity rather than SOX8 specifically, because *Sox8* and *Sox10* share a binding motif and act jointly to maintain myelin gene expression (Turnescu et al. 2018); the reduction of SOX8 protein within OLIG2+ cells provides the paralogue‐specific evidence.

In POSA mice we observed a highly ramified microglial morphology, which has also been observed in an adult sleep apnea model (Aviles‐Reyes et al. 2010) and is consistent with a chronic inflammatory state (Kim et al. 2024). Inflammation could contribute to cognitive deficits in POSA through two distinct mechanisms. First, microglial activation could drive the loss of neural stem cells and suppressed neurogenesis found in this POSA model (Chandrakantan et al. 2025). Indeed, past work demonstrated that hippocampal inflammation directly suppresses hippocampal neurogenesis (Ekdahl et al. 2003, 2009). Second, microglial activation could impair neuronal activity. Microglia–neuron interactions shape normal circuit function and changes in activation state can adversely affect neuronal activity, structure, and plasticity, manifesting as impaired cognitive function (Badimon et al. 2020; Shea and Villeda 2025). Notably, excessive microglial activation is observed in other neurodevelopmental disorders, including Down syndrome, where reducing inflammation has been shown to improve cognition (Pinto et al. 2020). Therefore, suppressing pathological inflammation in POSA could represent a therapeutic strategy.

Astrocytes closely interact with synapses and blood vessels to coordinate brain function. Numerous studies have identified the importance of a complex astrocyte morphology for proper regulation of synapse function and complex behaviors (Huang et al. 2020; Endo et al. 2022; Cheng et al. 2023; Baldwin et al. 2024; Williamson, Kwon, et al. 2025). Thus, the reduced complexity of hippocampal stratum moleculare astrocytes in POSA may dysregulate function of memory‐related circuits. Future studies should directly investigate how astrocyte‐neuron interactions change in POSA with causal manipulation experiments. Notably, a previous study observed increased astrocyte complexity in an adult sleep apnea model (Aviles‐Reyes et al. 2010). This difference highlights the importance of pediatric specific models, which may involve distinct pathology due to effects on sensitive developmental processes such as astrocyte morphogenesis (Williamson, Murali, and Deneen 2025). We also identified substantial changes in expression of astrocytic genes that encode endfoot proteins. Of these, we validated a protein level loss of the brain glucose transporter GLUT1 (*Slc2a1*). Notably, haploinsufficiency of the *SLC2A1* gene in humans causes a neurodevelopmental syndrome involving developmental delay and multifaceted impairments of cognitive abilities (Ito et al. 2015). Given the importance of glucose for all facets of brain function, the loss of GLUT1 in POSA could contribute to cognitive impairments. However, definitive mechanistic studies are needed to establish a link between altered astrocyte function and cognitive deficits in POSA.

Our results should be interpreted with limitations in mind. One limitation is that our POSA model focuses on intermittent hypoxia as the major determinant of cognitive impairments. While human data suggest that hypoxic episodes are directly linked to worsened cognition (Chandrakantan et al. 2020, 2025), incorporating other aspects of the disease such as sleep fragmentation and sleep arousal may provide a more complete POSA model. Next, it remains unclear how intermittent hypoxia drives transcriptional changes across cell types. Future studies could examine hypoxia‐sensitive pathways including hypoxia‐inducible factor signaling, receptor for advanced glycation end product signaling, and oxidative stress. Finally, it is important to recognize the descriptive nature of our single‐nucleus RNA‐seq analyses. While we have identified multiple cellular and molecular changes in this POSA model, future studies will need to perform direct manipulations of gene candidates to demonstrate causal relationships.

The transcriptomic analysis is also limited by the small number of biological replicates. Pseudobulk analyses showed strong directional concordance with the cell‐level MAST estimates, but only a subset of genes reached sample‐level FDR thresholds; modest‐effect DEG calls should therefore be considered discovery‐level pending validation in larger cohorts. A processing sub‐batch was also partially confounded with condition, preventing either analysis from fully separating condition from potential processing effects. Accordingly, our interpretation emphasizes concordant effect estimates across methods rather than the absolute number of significant genes. Importantly, our in vivo validation of expression changes of target genes and proteins was highly concordant across snRNA‐seq, qPCR, western blot, and immunostaining. The use of multiple validation approaches with consistent results provides strong support for the utility of our snRNA‐seq dataset.

Overall, our study provides new insights into the mechanisms underlying cognitive impairments in POSA. The extent of cellular and molecular changes across non‐neuronal cell types suggests that broad dysfunction of glial cells is a previously unappreciated aspect of POSA pathology that may drive cognitive deficits. Targeting the cellular and molecular changes we have identified may yield useful therapeutic approaches for children with POSA.

## Author Contributions

M.R.W., J.B., H.‐H.J., and A.C. conceived of and designed experiments. M.R.W, J.B., M.J.H., T.Z., A.S.H., C.J.Y., K.N., R.A., A.C.A., and A.C. performed experiments and data analysis. M.R.W., F.K., H.K.L., A.S.H., B.D., H.‐H.J., and A.C. supervised the work. M.R.W., J.B., and A.C. wrote the paper. All authors edited the paper.

## Funding

This work was supported by American Heart Association (25POST1363224), NIH R35 (NS132230), and NIH K08 (HL161263).

## Conflicts of Interest

The authors declare no conflicts of interest.

## Supporting information


**Figure S1:** Additional feature plots showing marker gene expression specificity.
**Figure S2:** Gene ontology biological process analysis of differentially expressed genes in neuron subtypes. (A) GO analysis of genes upregulated in excitatory neurons. (B) GO analysis of genes downregulated in excitatory neurons. (C) GO analysis of genes upregulated in granule cell neurons. (D) GO analysis of genes downregulated in granule cell neurons. (E) GO analysis of genes downregulated in inhibitory neurons.
**Figure S3:** Differentially expressed genes shared across broad cell types between control and POSA samples. (A) UpSet plot showing the distribution and intersections of genes downregulated in POSA across broad cell types. (B) UpSet plot showing the distribution and intersections of genes upregulated in POSA across broad cell types. Horizontal bars indicate the total number of differentially expressed genes per cell type, and vertical bars indicate the sizes of intersections for the indicated combinations of cell types.
**Figure S4:** Pseudobulk sensitivity analysis of single‐nucleus differential expression. (A, B) Sample‐level log2 fold changes from pseudobulk edgeR‐QLF and DESeq2 analyses (three control versus three POSA biological replicates per population) compared with cell‐level MAST log2 fold changes, pooled across clusters. The dashed line denotes identity; Spearman *ρ* and the number of assessed gene‐cluster pairs are shown. (C) Overlap of genes reaching FDR < 0.10 with edgeR and DESeq2.
**Figure S5:** Validation of target gene RNA expression with qPCR. Plots show normalized RNA expression of genes indicated at the top of each graph. *p < 0.05, **p < 0.01, ***p < 0.001; Welch's corrected *t* ‐tests. *n* = 4–8 mice per group; group sizes are indicated by the number of dots. Data are presented as mean ± SEM.
**Figure S6:** Western blot validation of molecular target expression. (A) Representative western blots for target proteins and β‐actin control. Molecular weight ladder is shown on the left. (B) Quantification of protein expression from western blots. *p* values are noted on each graph. All graphs share the same *y*‐axis title. Two‐tailed *t* ‐tests. *n* = 6 mice per group. (C) Representative western blot and quantification for GLUT3. *p* = 0.803, two‐tailed *t* ‐test. *n* = 6 mice per group. GLUT3 served as a negative control since *Glut3* RNA was not differentially expressed in the snRNA‐seq dataset.
**Figure S7:** Loss of NDUFS4 in subgranular zone neural stem cells in POSA. (A) Representative images showing reduced NDUFS4 within GFAP+ neural stem cells within the subgranular zone. (B) Quantification of NDUFS4+ GFAP+ cells within the subgranular zone. **p = 0.0067, Welch's corrected *t* ‐test. *n* = 3 mice per group. FOV = field of view.
**Figure S8:** Subclustering analysis of oligodendrocyte‐lineage cells. (A) Subclustering and feature plots of oligodendrocyte‐lineage cells. (B) Signature scores of oligodendrocyte‐lineage subclusters.
**Figure S9:** The *Sox8* and *Sox10* regulons are not separable by SCENIC. (A) Per‐nucleus AUCell activity of the two regulons across all 153,707 nuclei. (B) Overlap of their target gene sets. (C) Both regulons peak in ODC2 across the oligodendrocyte lineage. (D) Both decrease in ODC2 in POSA.
**Figure S10:** Regions examined and additional images of oligodendrocyte and astrocyte morphology. (A) Image of white matter (corpus callosum) and gray matter (stratum moleculare) regions analyzed. (B) Representative images of tdTomato+ oligodendrocytes in the corpus callosum dorsal to the hippocampus. Oligodendrocytes are labeled with *NG2‐CreER; Rosa‐LSL‐tdTomato* mice. (C) Representative images of GFP+ astrocytes in the dentate gyrus stratum moleculare. Cropped images are shown in Figure 7. Astrocytes are labeled with *Aldh1l1‐GFP* mice.
**Figure S11:** Subclustering analysis of microglia. (A) Subclustering and feature plots of microglia. (B) Signature scores of microglia subclusters.
**Figure S12:** Additional analysis of microglia morphological feature data. (A) Confusion matrix showcasing the classification performance of the random forest model used to classify between healthy and POSA reconstructed microglia. The matrix is the sum of across 5 cross‐validations. (B) Mean ± SD precision and recall of the random forest model for healthy versus POSA classes across 5 cross‐validations. (C) Comparison of the absolute value of feature importance metrics, Gini and SHAP, ranking the importances of microglia morphology features for the random forest model to predict a sample as healthy or POSA. (D) SHAP plot ranking the importance of microglia morphology features and their value magnitudes for the random forest model to predict a sample as healthy or POSA. (E) Reconstructions of healthy and POSA microglia, showcasing their structural differences.

**Table S1:** Pseudobulk threshold sensitivity analysis.

**Table S2:** Primer sequences.

**Table S3:** Definitions of subcluster acronyms.

**Table S4:** Pseudobulk significant DEGs.

## Data Availability

All raw snRNA‐seq data are available through the NCBI Gene Expression Omnibus with accession number GSE319173. Code for training the random forest model is available at https://github.com/cjy8s/Glial‐Dysfunction‐POSA. Glial cell morphology data, pre‐ and post‐processing, and analysis are available upon reasonable request.
