# Supplementary material for: Glial Dysfunction and Memory Impairments in a Model of Pediatric Obstructive Sleep Apnea
Source: Glia. 2026 Sep 18;74(11):e70230. doi: 10.1002/glia.70230 (PMC13589049; doi:10.1002/glia.70230)
Supplement: Supplementary file 1 [file GLIA-74-0-s001.docx]

**Supplemental Table 2**. Primer sequences.

| Primer | Sequence |
| --- | --- |
| Sox2-F | GCGGAGTGGAAACTTTTGTCC |
| Sox2-R | CGGGAAGCGTGTACTTATCCTT |
| Lrrk2-F | ATCTCACCCTTCATGCTTTCTG |
| Lrrk2-R | TCTCAGGTCGATTGTCTAAGACT |
| Sox8-F | CGAGGGGATACTGCTGAGG |
| Sox8-R | AGCTCTGCGTTATGGAGATGC |
| Qdpr-F | GTGCCAGCGTGGTTGTTAAG |
| Qdpr-R | GCTTCCACATCATGTCACAGTT |
| MBP-F | GGCCAGTAAGGATGGAGAGAT |
| MBP-R | CCTCTGAGGCCGTCTGAGA |
| Tfe3-F | CGAGCCGTGTTCCTGCTATT |
| Tfe3-R | TGCAGTGATATTGGGAGGCTG |
| Glut1-F | CAGTTCGGCTATAACACTGGTG |
| Glut1-R | GCCCCCGACAGAGAAGATG |
| Ndufs4-F | CTGCCGTTTCCGTCTGTAGAG |
| Ndufs4-R | TGTTATTGCGAGCAGGAACAAA |
| GAPDH-F | AGCCTCGTCCCGTAGACAAAA |
| GAPDH-R | TGGCAACAATCTCCACTTTGC |
