# Supplementary material for: Glial Dysfunction and Memory Impairments in a Model of Pediatric Obstructive Sleep Apnea
Source: Glia. 2026 Sep 18;74(11):e70230. doi: 10.1002/glia.70230 (PMC13589049; doi:10.1002/glia.70230)
Supplement: Supplementary file 2 [file GLIA-74-0-s003.docx]

**Supplemental Figures**

**Supplemental Figure 1**. Additional feature plots showing marker gene expression specificity.

**Supplemental Figure 2**. Gene ontology biological process analysis of differentially expressed genes in neuron subtypes. A) GO analysis of genes upregulated in excitatory neurons. B) GO analysis of genes downregulated in excitatory neurons. C) GO analysis of genes upregulated in granule cell neurons. D) GO analysis of genes downregulated in granule cell neurons. E) GO analysis of genes downregulated in inhibitory neurons.

**Supplemental Figure 3**. Differentially expressed genes shared across broad cell types between control and POSA samples. A) UpSet plot showing the distribution and intersections of genes downregulated in POSA across broad cell types. B) UpSet plot showing the distribution and intersections of genes upregulated in POSA across broad cell types. Horizontal bars indicate the total number of differentially expressed genes per cell type, and vertical bars indicate the sizes of intersections for the indicated combinations of cell types.

**Supplemental Figure 4.** Pseudobulk sensitivity analysis of single-nucleus differential expression. A,B) Sample-level log2 fold changes from pseudobulk edgeR-QLF and DESeq2 analyses (three control versus three POSA biological replicates per population) compared with cell-level MAST log2 fold changes, pooled across clusters. The dashed line denotes identity; Spearman ρ and the number of assessed gene-cluster pairs are shown. C) Overlap of genes reaching FDR < 0.10 with edgeR and DESeq2.

**Supplemental Figure 5.** Validation of target gene RNA expression with qPCR. Plots show normalized RNA expression of genes indicated at the top of each graph. *P < 0.05, **P < 0.01, ***P < 0.001; Welch’s corrected t tests. n = 4-8 mice per group; group sizes are indicated by the number of dots. Data are presented as Mean ± SEM.

**Supplemental Figure 6.** Western blot validation of molecular target expression. A) Representative western blots for target proteins and β-actin control. Molecular weight ladder is shown on the left. B) Quantification of protein expression from western blots. P values are noted on each graph. All graphs share the same y-axis title. Two-tailed t tests. n = 6 mice per group. C) Representative western blot and quantification for GLUT3. P = 0.803, two-tailed t test. n = 6 mice per group. GLUT3 served as a negative control since *Glut3* RNA was not differentially expressed in the snRNA-seq dataset.

**Supplemental Figure 7**. Loss of NDUFS4 in subgranular zone neural stem cells in POSA. A) Representative images showing reduced NDUFS4 within GFAP+ neural stem cells within the subgranular zone. B) Quantification of NDUFS4+ GFAP+ cells within the subgranular zone. **P = 0.0067, Welch’s corrected t test. n = 3 mice per group. FOV = field of view.

**Supplemental Figure 8**. Subclustering analysis of oligodendrocyte-lineage cells. A) Subclustering and feature plots of oligodendrocyte-lineage cells. B) Signature scores of oligodendrocyte-lineage subclusters.

**Supplemental Figure 9**. The *Sox8* and *Sox10* regulons are not separable by SCENIC. A) Per-nucleus AUCell activity of the two regulons across all 153,707 nuclei. B) Overlap of their target gene sets. C) Both regulons peak in ODC2 across the oligodendrocyte lineage. D) Both decrease in ODC2 in POSA.

**Supplemental Figure 10**. Regions examined and additional images of oligodendrocyte and astrocyte morphology. A) Image of white matter (corpus callosum) and gray matter (stratum moleculare) regions analyzed. B) Representative images of tdTomato+ oligodendrocytes in the corpus callosum dorsal to the hippocampus. Oligodendrocytes are labeled with *NG2-CreER; Rosa-LSL-tdTomato* mice. C) Representative images of GFP+ astrocytes in the dentate gyrus stratum moleculare. Cropped images are shown in Figure 7. Astrocytes are labeled with *Aldh1l1-GFP* mice.

**Supplemental Figure 11**. Subclustering analysis of microglia. A) Subclustering and feature plots of microglia. B) Signature scores of microglia subclusters.

**Supplemental Figure 12**. Additional analysis of microglia morphological feature data. A) Confusion matrix showcasing the classification performance of the random forest model used to classify between healthy and POSA reconstructed microglia. The matrix is the sum of across 5 cross-validations. B) Mean +/- SD precision and recall of the random forest model for healthy versus POSA classes across 5 cross-validations. C) Comparison of the absolute value of feature importance metrics, Gini and SHAP, ranking the importances of microglia morphology features for the random forest model to predict a sample as healthy or POSA. D) SHAP plot ranking the importance of microglia morphology features and their value magnitudes for the random forest model to predict a sample as healthy or POSA. E) Reconstructions of healthy and POSA microglia, showcasing their structural differences.

Supplementary Table 1: Pseudobulk threshold sensitivity analysis.

Supplementary Table 2: Primer sequences.

Supplementary Table 3: Definitions of subcluster acronyms.

Supplementary Table 4: Pseudobulk significant DEGs.
