# Supplementary material for: Glial Dysfunction and Memory Impairments in a Model of Pediatric Obstructive Sleep Apnea
Source: Glia. 2026 Sep 18;74(11):e70230. doi: 10.1002/glia.70230 (PMC13589049; doi:10.1002/glia.70230)
Supplement: Supplementary file 3 [file GLIA-74-0-s004.docx]

**Supplemental Table 3**. Definitions of subcluster acronyms.

| Abbreviation | Definition |
| --- | --- |
| GC | Granule cell |
| OPC | Oligodendrocyte progenitor cell |
| AST | Astrocyte |
| EC | Endothelial cell |
| ExN | Excitatory neuron |
| FB | Fibroblast |
| InN | Inhibitory neuron |
| MGL | Microglia |
| ODC | Oligodendrocyte |
| SC | Stem cell |
